# Subsets of Visceral Adipose Tissue Nuclei with Distinct Levels of 5-Hydroxymethylcytosine

**DOI:** 10.1371/journal.pone.0154949

**Published:** 2016-05-12

**Authors:** Ping Yu, Lexiang Ji, Kevin J. Lee, Miao Yu, Chuan He, Suresh Ambati, Elizabeth C. McKinney, Crystal Jackson, Clifton A. Baile, Robert J. Schmitz, Richard B. Meagher

**Affiliations:** 1 Department of Genetics, University of Georgia, 120 East Green Street, Athens, GA, 30602, United States of America; 2 Institute of Bioinformatics, University of Georgia, 120 East Green Street, Athens, GA, 30602, United States of America; 3 GRU-UGA Medical Partnership, University of Georgia Health Sciences Campus, Prince Avenue, Athens, GA, 30602, United States of America; 4 Department of Chemistry, University of Chicago, 5735 S Ellis Ave, Chicago, IL, 60637 USA; 5 Abeome Corporation, Athens, GA, 111 Riverbend Road, 30602, United States of America; 6 Department of Foods and Nutrition, University of Georgia, 305 Sanford Dr, Athens, GA, 30602, United States of America; New England BioLabs, Inc, UNITED STATES

## Abstract

The reprogramming of cellular memory in specific cell types, and in visceral adipocytes in particular, appears to be a fundamental aspect of obesity and its related negative health outcomes. We explored the hypothesis that adipose tissue contains epigenetically distinct subpopulations of adipocytes that are differentially potentiated to record cellular memories of their environment. Adipocytes are large, fragile, and technically difficult to efficiently isolate and fractionate. We developed fluorescence nuclear cytometry (FNC) and fluorescence activated nuclear sorting (FANS) of cellular nuclei from visceral adipose tissue (VAT) using the levels of the pan-adipocyte protein, peroxisome proliferator-activated receptor gamma-2 (PPARg2), to distinguish classes of PPARg2-Positive (PPARg2-Pos) adipocyte nuclei from PPARg2-Negative (PPARg2-Neg) leukocyte and endothelial cell nuclei. PPARg2-Pos nuclei were 10-fold enriched for most adipocyte marker transcripts relative to PPARg2-Neg nuclei. PPARg2-Pos nuclei showed 2- to 50-fold higher levels of transcripts encoding most of the chromatin-remodeling factors assayed, which regulate the methylation of histones and DNA cytosine (e.g., *DNMT1*, *TET1*, *TET2*, *KDM4A*, *KMT2C*, *SETDB1*, *PAXIP1*, *ARID1A*, *JMJD6*, *CARM1*, *and PRMT5*). PPARg2-Pos nuclei were large with decondensed chromatin. TAB-seq demonstrated 5-hydroxymethylcytosine (5hmC) levels were remarkably dynamic in gene bodies of various classes of VAT nuclei, dropping 3.8-fold from the highest quintile of expressed genes to the lowest. In short, VAT-derived adipocytes appear to be more actively remodeling their chromatin than non-adipocytes.

## Introduction

There is a critical need to perform cell-type-specific epigenetic analyses of adipocytes within adipose tissues, because of their likely direct role in obesity and its comorbidities, including tissue inflammation, many cancers, cardiovascular disease, Type II Diabetes, and Alzheimer’s. Epigenetic controls function at the level of specific cell types, and yet, the vast majority of published epigenetic studies examine chromatin structures (i.e., epitypes) of whole organs or tissues (e.g., adipose tissue) and most commonly whole blood. These aggregated results from mixtures of cell types, however, do not accurately capture the real biology of specific cell types, which is essential to understand for designing improved therapies. For example, Reinius et al., 2012 [1] examined the DNA cytosine methylation profiles for seven purified blood leukocyte cell types in healthy individuals. They found that the DNA methylation profile (epitype) of each cell type varied significantly from that of whole blood. Pairwise comparisons of the seven leukocyte types varied at 9.5% to 40% of the 485,000 cytosine methylation sites assayed. Because the epitype of peripheral white blood cells is the weighted average of methylation differences among cell types, whole blood data has relatively weak statistical significance. The results from two studies examining the link between gene-specific DNA cytosine methylation at CG dinucleotides and systemic lupus erythematosus (SLE) highlight the need for cell- type-specific analyses. While whole blood DNA produced median p values of 10^−3^ for some methylation sites associated with SLE, the methylation data from CD4+ T cells resulted in median p values of 10^−7^ for numerous sites associated with SLE [2,3]. Hence, most of these latter data are statistically and most likely biologically significant. Our technical goal herein was to enable the cell-type-specific epigenetic analysis of adipocyte populations from within adipose tissues, and thereby obtain more biologically relevant data on chromatin structures.

The diversity of cell types in adipose tissue complicates attempts at cell-type-specific analysis. In addition to various classes of preadipocytes as well as maturing and mature adipocytes, adipose tissue is rich with blood vessels, endothelial cells and numerous lymphoid cell types (e.g., T cells, neutrophils, and natural killer cells) [4–9]. Furthermore, obese adipose tissue cell populations are different from lean ones. The pathology of obesity results in more mesenchymal stem cells (MSCs) being directed to develop into white adipocytes and the enlargement of existing adipocytes [7,10–13]. Larger numbers of T lymphocytes and neutrophils infiltrate adipose tissue in obese individuals relative to lean individuals [14–16]. Immunocytochemistry reveals individual obese adipocytes surrounded by several-fold larger numbers of inflammatory leukocytes than surrounding lean adipocytes [4–6]. Because any analysis of chromatin modification from adipose tissue would represent a weighted average of the various cell types, changes in the ratios of adipocyte subpopulations and non-adipocyte cell types would compromise conclusions about epigenetic reprogramming and disease.

White adipocytes may be enzymatically dissociated from adipose tissues, but they are difficult to isolate efficiently because of their large size (50 to 200 μm) and tendency to lyse rapidly during manipulation or brief storage [17]. Fluorescence activated cell sorting (FACS) has been used to fractionate dissociated adipocytes, but the large cell sizes require special instrumentation to prevent clogging and slow flow rates [18]. Furthermore, very few immunochemical surface markers are available that distinguish subsets of adipocytes. Several technical approaches have been applied to increase the cell-type-specificity of epigenetic studies for cell types that are difficult to isolate, but these too have their limitations. For example, great improvements have been made in laser-capture micro-dissection (LCM) technologies, yet, relatively small numbers of cells are recovered and tissue processing can be time consuming and expensive [19–21]. Model organisms may be engineered for the Isolation of Nuclei Tagged in Specific Cell Types (INTACT), but transgenic animals are expensive to make and maintain and INTACT cannot be applied diretly to isolate human cellular nuclei [22,23]. As an alternative, Fluorescent Nuclear Cytometry (FNC) and Fluorescence Activated Nuclear Sorting (FANS) have been used to compare the transcriptome, epigenome, and proteome among classes of brain cellular nuclei [24–27]. For example, using FANS we recently characterized a subset of neuronal cellular nuclei that were decondensed and expressed exceptionally high levels of stem cell and cell cycle neurotrophic, synaptotropic, and chromatin modifying factors markers, relative to the majority of neuronal and non-neuronal nuclei [28]. Overexpression of this machinery may be associated with the rapid turnover of chromatin modifications in cell types most likely to be potentiated to respond to their environment and more rapidly record cellular memories [29]. Using FNC and FANS to study cellular nuclei as surrogates for isolated cells is still in its infancy, but these technologies are relatively simple to employ for “problematic tissues” and have the potential to reveal a great deal about epigenetically distinct cell populations within adipose tissues.

There is mounting evidence that adipocytes within adipose tissues may be epigenetically programmed in response to obesity, obesity-related diseases, exercise, diet, and sleep, as well as when adipocytes exit from the cell cycle and proceed through adipogenesis [30–36]. Our long-term working hypothesis is that adipose tissue contains epigenetically distinct subpopulations of adipocytes, which are differentially potentiated to record cellular memories. However, there is currently limited information about subpopulations of adipocytes from within tissue or their mechanisms of cellular memory. A number of nucleosomal histone side chain modifications as well as modifications to DNA cytosine residues are correlated with the adipogenic program and postulated to play a role in programming preadipocytes and mature adipocytes (see Discussion). Of particular interest is the recent evidence that gene-region- and enhancer-region-specific 5-hydroxymethylcytosine (5hmC) at CG dinucleotides may define genes poised to change their expression or already having increased expression in part through localized loss of 5mC [37,38]. During the *in silico* differentiation of 3T3-L1 preadipocytes to adipocytes there is 2-fold increase in 5hmC levels in activated vs. repressed gene regions and as much as a 10-fold increase in 5hmC in the fatty acid binding protein 4, *FABP4* gene [39]. Hence, the oxidation of 5mC to 5hmC is strongly associated with adipogenesis.

**Fig 1:**
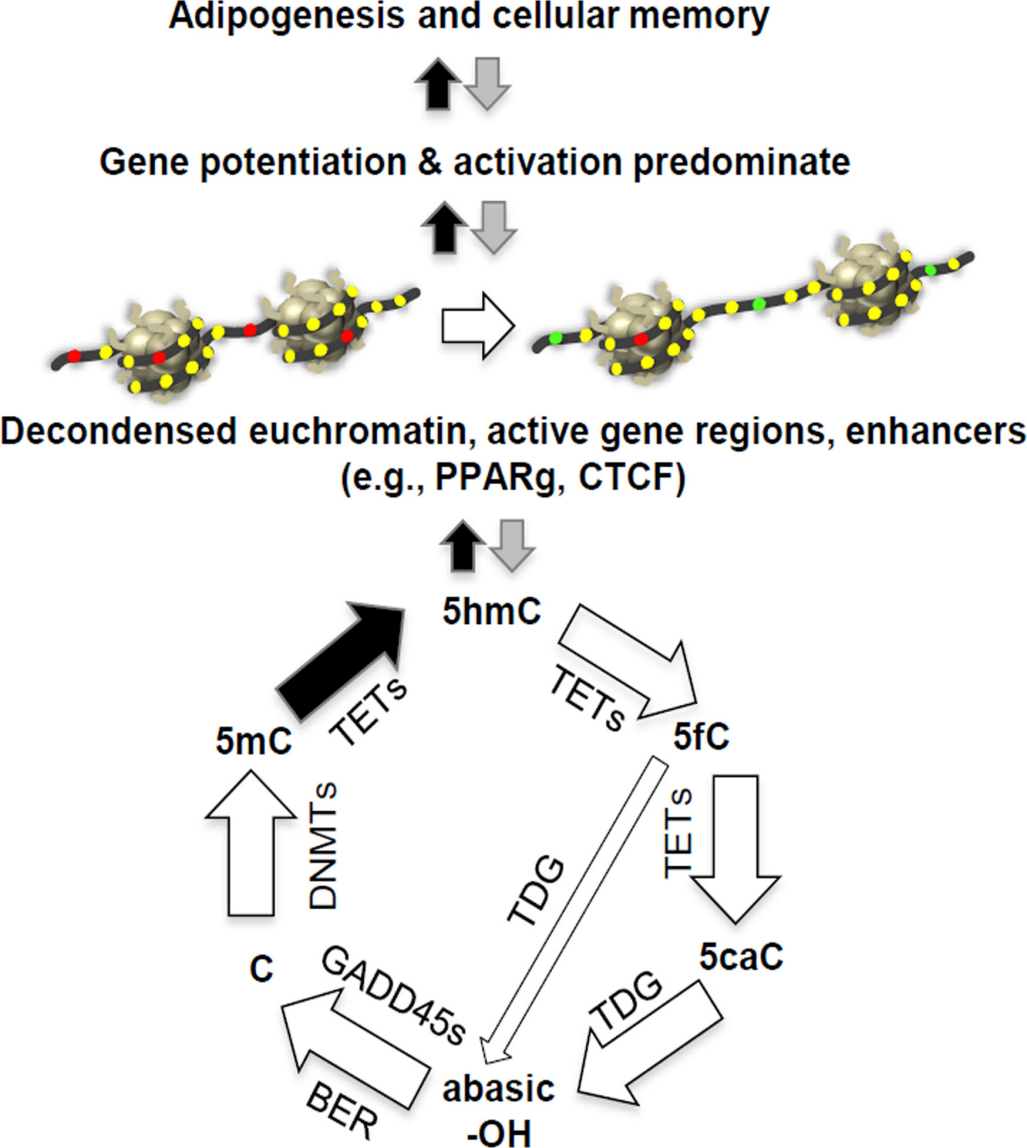
Turnover cycle for DNA cytosine modification at CG dinucleotides and its potential impact on adipose tissue (Diagram modified from Dubois-Chevalier, 2015 and Kohl, 2013). A model is suggested in which the dynamic modification cycle of DNA cytosine residues (C) is linked to ubiquitous (CTCF) and adipocyte-specific (PPARg) transcription factor enhancement of gene expression during adipogenesis and in mature adipocytes. CTCF and PPARg recruit TET enzymes to promote 5mC hydroxymethylation and activate transcription of PPARg. The lower panel shows the cyclic turnover of modified cytosine (C) residues and emphasizes that TETs catalyze the rate-limiting step of removing 5mC by oxidation to 5-hydroxymethylcytosine (5hmC). TET activity further oxidizes 5hmC to 5-formalcytosine (5fC) and 5-carboxycytosine (5caC). The essential roles of other factors include DNMTs in the methylation of C to 5-methylcytosine (5mC), thymine DNA glycosidase (TDG) and methyl-CG binding domain protein 4 (MBD4) in the excision of 5fC or 5caC by creating a single nucleotide gap, and gap repair back to a C residue by base excision repair (BER) machinery such as the GADD45s. The gene-region-specific balance of these activities determines the levels of C, 5mC, and 5hmC. The diagram was modified from those in previous publications [41,115].

The cyclic turnover of 5´-modified cytosine is summarized (see Fig 1). DNA methyltransferases (DNMTs) methylate DNA cytosine to 5mC, while Ten-eleven translocation methylcytosine dioxygenases (TETs) catalyze its conversion to 5hmC and to other more oxidized forms (5fC, 5caC). Although, 98% of TET activity is restricted to modified cytosine residues in the CG dinucleotide context. Thymine-DNA glycosylase (TDG) acts on 5fC or 5caC to generate an abasic site (-OH). The base excision repair pathway (BER) and factors like the GADD45s recognize a G residue in the antiparallel DNA strand and restore cytosine. In general, 5hmCG dinucleotides mark a small subset of antiparallel CGs, which may or may not also be 5mC modified (5mCGs) in the antiparallel strand, such that increases in one of these two modifications at a site is not always correlated with the loss of the other. TET dioxygenase-catalyzed oxidation of 5mC to 5hmC at constitutive CTS (CTCF binding sites) and PPARg enhancers (PPAREs) appears to be part of and perhaps may be essential to adipogenesis [40,41]. The ADP-ribose polymer attached to parylated-PPARg binds TET enzymes to catalyze the localized conversion of 5mC to 5hmC [42], which begins to outline a mechanism connecting 5hmC modification and adipogenesis. Enhancer cytosine hydroxymethylation appears to be tissue-specific, where it acts on adipocyte-specific enhancers during a 3T3-L1 cell’s differentiation to an adipocyte and on neuronal-specific enhancers during neurogenesis in a cultured neural progenitor cell type [39]. Little is known about the precise role of gene-region distribution and changes in 5hmC in adipocytes, although in neurons it has been proposed that high levels of gene-region 5hmC “creates pre-modified sites that are poised for subsequent demethylation and activation at a later developmental stage” prepared for “on demand gene regulation” [38,43].

Initial pioneering studies in situ suggest that large-scale gene silencing by DNA methylation might be essential to the commitment to adipogenesis. In particular, using 5´-deoxyazycytidine to inhibit DNMT catalyzed cytosine methylation during the contact inhibition and licensing (i.e., specification) stage prior to differentiation causes a severe reduction in the efficiency of subsequent adipogenesis [44]. After licensing and the addition of a differentiation cocktail of growth factors there is a brief period of mitotic clonal expansion. The differentiation to lipid body-rich mature adipocytes proceeds after preadipocytes exit the cell cycle [44]. Two days after the differentiation of 3T3-L1 cells begins global DNA cytosine methylation levels increase, as do 5mC levels at the *CEBPA* promoter region [44]. DNMT1 levels increase rapidly during the first 24 hours after inducing differentiation [45] and decline later as mature adipocytes are formed. But DNMT1 is defined as a maintenance enzyme, not a de novo methyltransferase. Hence, DNMT1 levels may account for the increase in 5mC by more efficiently maintaining methylation and by supporting increases in de novo methylation. Small interfering RNA silencing of the de novo cytosine methyltransferase DNMT3a in 3T3-LI preadipoctyes significantly blocks adipogenesis [44] emphasizing a positive role for DNA methylation. However, additional work stands against this simple positive role for increased global 5mC levels in adipogenesis. Small RNA silencing of the maintenance methyltransferase DNMT1 in 3T3-L1 preadipocytes accelerates adipogenesis [45]. In addition, treating bone marrow derived MSCs, a normal precursor of adipocytes, with 5-azacytidine (5-azaC), a cytidine analog and inhibitor of methyltransferases, decreases both cell proliferation and differentiation into adipocytes and results in concomitant down-regulation of PPARg [46]. Finally, treating atrial cardiac cells with 5-azaC, an inhibitor of all DNMT activity, reduces 5mC to produce an interesting outcome, wherein these cells trans-differentiate into lipid body-containing adipocytes [47]. Among the likely possibilities that might explain these complex results, the starting epitype of the progenitor preadipocyte cell undoubtedly affects their developmental potential, as does their existing chromatin modification machinery. Additionally, the C-residue sequence specificity and regulation of the cytosine modification cycle will affect the genes being altered and the developmental outcome. Hence, the role of the DNA cytosine modification cycle appears distinct during stem cell differentiation into preadipoctyes, during adipogenesis, and in mature adipocytes. The roles of cytosine modification in adipogenesis are more complex than simply removing its silencing effects on appropriate adipogenic gene-regions and enhancers. In any case, we focused on the formation of 5hmC, because it appears to be the rate-limiting step in removing 5mC at CG dinucleotides and hence rate limiting to the turnover of modified cytosine.

Herein, we extend FNC and FANS to the analysis of adipocyte nuclei within adipose tissue. We developed techniques to rapidly isolate cellular nuclei from fixed adipose tissue, such that both nuclear structure and chromatin modification would be preserved. We showed that cytometry was easily applied to characterize subpopulations of adipose tissue nuclei. Nuclear sorting identified subpopulations of adipocyte and non-adipocyte nuclei that differentially expressed a significant fraction of the epigenetic machinery we assayed. Adipocyte nuclei were identified that had highly elevated levels of factors involved in the regulation of histone methylation and DNA cytosine modification, and in particular displayed widely divergent levels of 5-hydroxymethylcytosine (5hmC) across the gene body of different groups of genes.

## Materials and Methods

### *Sus scrofa* tissues

Animals (*Sus scrofa* 6 month old, 220–280 lbs) were slaughtered at UGA’s abattoir, which is a USDA licensed facility (Establishment #7421A). All institutional and USDA guidelines for the care and use of animals were followed. Fresh kidney-associated visceral adipose tissue (VAT) was harvested and chilled on ice for no more than 2 h prior to being processed to purify nuclei or flash frozen in liquid nitrogen and stored at -80°C until use. Pigs were hybrids from PIC line 29 sows and PIC line 337 semen.

### Protocol for isolating cellular nuclei from adipose tissue

The following rapid protocol for isolating cellular nuclei from adipose tissue is an extension of the simplified method described recently for brain cell nuclei [28]. Freshly dissected and minced adipose tissue was treated for 1 hour at RT to 2 months at 4°C in four volumes (w/v) of 0.3SPBSTA (0.3 M Sucrose in PBSTA, 20 mM KH_2_PO_4_, 20 mM Na_2_HPO_4_, pH = 7.2, 137 mM NaCl, 3.0 mM KCl, 0.1% Triton X 100, 0.1% sodium azide), plus 3.7% freshly added formalin. The protocol works on fresh tissue, but the yield of nuclei is lower than for fixed. The fixed tissue is heated briefly to 60°C to solubilize the fat (5 to 10 min depending upon the sample size). Typically 50 g of tissue was homogenized in a prewarmed (60°C water) Polytron (Fischer Sci) for 2.5 min at a setting of 6.5 in 8 volumes (w/v) of 0.3SPBSTA. The homogenate was filtered through large pieces (10 in. sq.) of Miracloth (Calbiochem, #475855) stretched loosely over a funnel. This filtration step prevented nuclei from being trapped with large pieces of cytoplasmic debris during the subsequent centrifugation and increased the yield of nuclei several fold. The filtrate was placed in centrifuge bottles or tubes and under-layered with 0.25 volumes of 1.4 M sucrose in SPBSTA. Nuclei were centrifuged in a pre-chilled rotor (4°C) through the sucrose cushion at 3,000xg for 20 min. The supernatant was removed gently by pouring it out from one of two holes made through the hardened fat layer. The nuclear pellet under the fat floatation pellet was gently re-suspended in with 0.3M SPBSTA. 3–5 ml aliquots were pressed slowly through 25 mm diameter Swinnex Nylon Net Filters with a 41-μm pore size (EMD Millipore). The yield of nuclei from freshly fixed VAT tissue was approximately 1.0 x 10^6^ nuclei/g tissue and a few-fold less from frozen tissue fixed subsequent to thawing. Nuclei were stored for up to one year at 4°C in PBSTBA (PBS + 0.1% Triton X-100 + 5% BSA + 0.02% Azide) and freshly added 4% formaldehyde. Storage did not seem to alter the quality of immunofluorescence staining for several markers assayed, but storage for more than three months did lower the yield of RNA. All reagents were purchased from Thermo Fisher (Waltham, MA), unless stated otherwise. In various experiments, this protocol has been scaled from 50 g to 50 mg of VAT, and these differed only in that the heat treatment may be omitted prior to homogenizing very small samples. This isolation protocol worked similarly, but with slightly lower yield than when using fresh unfixed or -80°C frozen adipose tissue that was immediately fixed after thawing. The protocol has worked as well with rat and mouse VAT, SAT, and BAT as it did with porcine VAT and SAT.

### Western blot analysis

Protein was extracted and resolved on SDS-PAGE gels as descried previously for brain nuclei [28]. Equal loading of total protein amounts in adipose tissue homogenates (H) and enriched nuclear fractions (N) was predetermined by coomassie blue staining of equivalent samples electrophoresed into through the stacking gel and for a brief period into the resolving gel. Relative protein loading could not be quantified if the coomassie gel was run as long as it was for the western blot gel, because few of the bands aligned in the VAT homogenates and nuclear samples and many bands were too weak to be compared as noted previously for similar comparisons in brain [28].

### IFM, FNC, and FANS analysis of nuclei

Immunochemical labeling of nuclei followed exactly the protocol used for brain nuclei [28]. For FNC 100,000 to 400,000 nuclei were incubated in 200 μl blocking solution with primary antibodies (S1 Table) at dilutions of 1:100 to 1:500 w/v for 1 h at room temperature. For FANS, where as many as 100-times more total nuclei were labeled in small volumes, the antibody concentration was much higher and was estimated based on the number of nuclei being examined (0.5 to 1.0 μg antibody per 10^6^ nuclei) and not based on the volume of buffer. In a typical FANS experiment, 15 μg of rabbit polyclonal antibody to PPARg2 (ab45036) was incubated with 20 x10^6^ nuclei in 500 μl blocking solution for 1 hr. After 3 washes with PBSTBA samples were co-stained with DAPI or propidium iodide (PI) at 20 μg/ml for 30 min. Photographic images of nuclei and tissue sections were made on a Leica TR600 epifluorescence microscope using a Hamamatsu ORCA-CR camera and Hamamatsu SimplePCI Image Analysis software to process images and measure nuclear areas and fluorescence intensities.

FNC and FANS were conducted as described previously [28]. The nuclear population was first gated for size and shape (S1A Fig) and DNA content (S1B Fig) to reduce the number of contaminating particles sorted. The fraction of 4C nuclei appeared low to undetectable in most VAT nuclear preparations, but there was a large percentage of nuclei that showed higher than 2C staining with DAPI. This signal may result from decondensed nuclei that have a very high RNA content, because DAPI has a modest fluorescence enhancement with dsRNA [48] as was observed for decondensed brain nuclei [28]. No significant population of doublet nuclei was detected during sorting (S1C Fig), and therefore, a doublet gate was not applied so as not to discriminate against large decondensed nuclei [28]. Furthermore, a pulse-width gate was not applied because of the concern that it might eliminate some very large decondensed nuclei that were of interest to this research. Figures of FNC and FANS data were prepared using FlowJo Software version 9.7.6 (Treestar, Inc. Ashland, Oregon).

### RNA, cDNA, and qRT-PCR

RNA from formalin-fixed nuclei was prepared and reverse transcribed into cDNA for qRT-PCR analysis as described previously [28]. Primers are listed in S2 Table. Multiple primer pairs were designed and assayed for each porcine target RNA and only those showing efficient amplification of product from total VAT RNA were selected. The primer pairs selected also had a product dissociation curve with only one peak (i.e., only one cDNA was amplified). Among several commonly used control transcripts that were examined [49], *beta-actin* and *RPL13* were relatively equivalently expressed among the four nuclear fractions, when qRT-PCR assays were normalized for equivalent cDNA input and suitable as endogenous controls. Each assay was run in triplicate and the Relative Quantity (RQ) of transcript was calculated based on the dCT method, including the standard deviation from the mean [50]. We were unable to find *bona fide MBD4* or *TDG* sequences in the porcine genome database, and hence their transcripts were not assayed by qRT-PCR.

### TAB-seq and Quintile Expression Data

#### DNA sample preparation

DNA was isolated from sorted PPARg2-High, pooled PPARg2-Med & -Low, and PPARg2-Neg porcine kidney VAT nuclei (~2 to 4 x 10^6^ nuclei per sample) using a DNeasy kit (Qiagen, Frederick, MD, USA #69504) according to the manufacturer’s recommendations. A heat treatment of 90°C for 1 h was included, after the proteinase K digestion, to hydrolyze off the formalin. DNA was quantified using a Qubit 2.0 Fluorometer (Invitrogen) with the Qubit dsDNA Assay Kit (Life Technologies # Q32853). TET-assisted bisulfite sequencing (TAB-seq) was performed as we previously described [51]. 0.5 ng of methyltransferase M.SssI methylated lambda DNA and 0.25 ng of 5hmC-containing pUC19 DNA were added per 1 μg of nuclear DNA prior to treatment as C/5mC/5hmC controls. 5hmC-containing pUC19 DNA was produced using PCR amplification with 5hmdCTP. After beta-GT-mediated glucosylation and Tet-mediated oxidation, the sequencing libraries were then prepared following the MethylC-seq protocol [52]. DNA sequencing was performed using an Illumina NextSeq500 Instrument at the University of Georgia’s Genomics Facility, with coverage estimated to range from 0.35 to 0.41 genome equivalents among the various samples (S3 Table) [53].

Due to high cost associated with deep coverage of the pig genome using WGBS, we chose an alternative strategy, looking instead at 5hmC metagene plots for hundreds to thousands of genes (groups of genes). Detailed 5mC data for the even larger maize genome were obtained using low-coverage whole-genome bisulfite sequencing and metagene plots [54]. To demonstrate that the metagene approach and our levels of coverage (i.e., 0.4X genome equivalents) for 5hmC were robust, we downloaded a published high-coverage (>13X genome equivalents) 5hmC dataset [38] for the mouse brain frontal cortex and then subsampled the number of reads ranging from 13X down to 0.2X genome equivalents of coverage. We then plotted 5hmC distribution for the six gene groups examined in this paper for each level of coverage. As can be seen in S2 Fig, coverage did not meaningfully alter the patterns of 5hmC distribution in metagene plots, even for coverage as low as 0.2X and for gene groups with as few as 60 genes.

#### TAB-seq data analysis

The raw sequence data were trimmed for adapters, preprocessed to remove low quality reads, aligned to the Sscrofa reference genome Sscrofa10.2 (GCA_000003025.4, http://www.ensembl.org/Sus_scrofa/) and analyzed as we described previously for TAB-seq analysis of 5hmC [55]. The reference genome assembly is based on DNA from a single Duroc pig, T J Tabasco. The control 5mC modified lambda DNA sequence was used to calculate the 5mC non-conversion rate upon Tet and bisulfite treatment. Non-CG dinucleotide sites were used to compute the non-conversion rate of unmodified cytosines upon bisulfite treatment (S3 Table). The 5hmC-containing pUC19 DNA was spiked in the genomic DNA as an internal control to evaluate the protection rate in the real samples (S3 Table). The protection rate is a measure of the percentage of 5hmCG that is protected from TET oxidation by using beta glucosyltransferase. This value is used to estimate true 5hmCG in the genome as it corrects for varying degrees of protection in this type of assay. For this analysis, only cytosines in the CG context were considered.

#### Quintiles expression data

We obtained extensive transcript expression data for porcine adipose tissue based on RNA-seq, covering a large dynamic range of expression levels [56]. Expression levels from the 16 adipose tissue samples presented were averaged to obtain a list of 25,321 expressed transcripts. Because in RNA-seq, the number of reads mapped to a gene is also a function of the total exonic length, the average expression level was divided by this exonic length of each gene to normalize expression levels. This list was broken into quintiles based on exon-normalized mRNA expression levels, resulting in “quintile of expression” gene lists with 5,064 to 5,065 genes in each list.

For each quintile of transcripts, the level of 5hmC was determined using weighted methylation level calculation [57] for each of 20 bins upstream, 20 bins within genes (between annotated TSS and TTS) and 20 bins downstream of genes. Each of the upstream and downstream bins spanned 5kb for a total of 100kb spanned in each direction. The within-gene regions, no matter what their length, were evenly divided among the 20 bins. Figures were prepared using ggplot2 [58].

### Availability of supporting data

The TAB-seq data set supporting the results of this article is available in NCBI GEO repository, accession number **GSE73684.** A unique persistent identifier and hyperlink to our dataset is http://www.ncbi.nlm.nih.gov/geo/query/acc.cgi?token=uridyaiefdidpan&acc=GSE73684.

### Statistical analysis

Bar graph data are presented with the mean ± standard error of the mean (SEM). The data for nuclear area and qRT-PCR were analyzed by one-way ANOVA with post hoc Tukey’s HSD test using Statistica software 7.1 (StatSoft; Tulsa, OK, USA). For particularly valuable statistical comparisons, the value of p<0.01 is denoted with * while a value of p<0.001 is denoted with **.

## Results

### Isolating and sorting adipocyte nuclei from adipose tissue

We considered possible nuclear protein markers that could be used to identify subsets of adipocyte nuclei. Members of the peroxisome proliferator-activated receptor (PPAR) subfamily of nuclear receptor transcription factors positively control transcription, the metabolism of glucose and lipids, and ultimately cell division and differentiation [59]. One of the three PPAR subtypes, PPAR gamma (PPARg) is induced during and is essential to adipogenesis, and it cooperates with many other factors in the process. PPARg binds to PPAR-enhancers and activates genes involved in adipocyte differentiation, lipid synthesis, and lipid storage [60]. While the best characterized subtype of PPARg protein, PPARg1, is strongly expressed in the nuclei of maturing and mature adipocytes (MMAs), it is also expressed in other cell types in adipose tissues including mesenchymal adipocyte lineage-committed cells, dedifferentiated fat cells, endothelial cells, and leukocytes (e.g., T-cells, neutrophils) [61–64]. Because of its essential roles in adipogenesis and lack of quantitative evidence to the contrary, we considered the possibility that PPARg1 protein might be most highly expressed in the most active adipocytes and therefore a good marker to quantitatively distinguish highly active adipocyte nuclei from the nuclei of other cell types.

**Fig 2:**
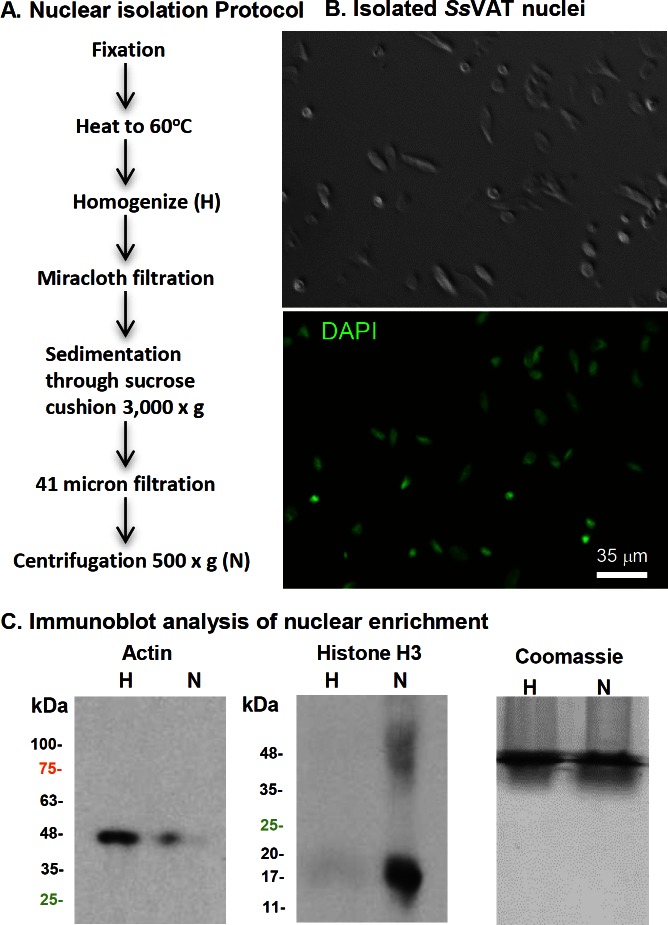
Purifying adipocyte cellular nuclei. **A.** Protocol for the rapid purification of cellular nuclei from adipose tissue. **B.** Phase contrast microscopic image (upper panel) and fluorescent microscopic image of DAPI staining (lower panel) of total VAT nuclei. DAPI stained for DNA (green). Phase contrast image of nuclei showed there was very little cellular debris. **C.** Western blot comparing the levels of actin and histone H3 in total *Ss*VAT homogenate (H) and purified *ss*VAT nuclear protein extracts (N).

A protocol for the rapid isolation of adipocyte nuclei was developed as outlined in Fig 2A and detailed in Materials and Methods. DIC microscopy of isolated *Ss*VAT nuclei and co-staining of DNA with DAPI showed that the enriched preparation of nuclei contained only modest amounts of cellular debris (Fig 2B). The method required only minor modifications from that for the isolation of brain cellular nuclei [28]. To further test the enrichment of nuclei, we compared proteins in crude VAT homogenate to proteins in purified nuclei using Western blotting (Fig 2C, 2H & 2N). The nuclear fraction (N) was highly enriched for nuclear protein histone H3 and the total VAT homogenate (H) was greatly enriched for the cytoplasmic protein actin. Our long-term interest was in the epigenetic alterations to chromatin structure, therefore, we focused on nuclei isolated from formalin-fixed fresh tissue.

We characterized nuclei that had been sorted based on PPARg1 levels. A relatively large fraction of all VAT nuclei stained strongly with antibodies to PPARg1, but there was a wide dynamic range in the staining consistent with the differential expression of PPARg1 among cell types (S3A Fig). Using qRT-PCR assays for cell-type-specific transcripts, we found that nuclei with higher levels of PPARg1 were not significantly enriched for adipocyte transcripts relative to transcripts marking other cell types (S4 and S5 Figs). For example, PPARg1-High nuclei had high levels of transcripts encoding IKAROS, a leukocyte-specific marker, higher than the levels in PPARg1-Neg nuclei. Note that the levels of most nuclear transcripts are strongly linearly correlated with their levels in total cellular RNA (R correlation coefficient = 0.94, supplementary data in Deal et al [22]). Hence, neither the cell-type-specificity nor the relative levels of PPARg1 protein expression were sufficient to identify subsets of adipocyte nuclei within adipose tissues.

An alternate upstream promoter and alternate RNA splicing produce a slightly longer isoform of PPARg, PPARg2, with a 28-amino-acid extension on the N-terminus relative to PPARg1 [65]. Although less well characterized than the shorter PPARg1 isoform, PPARg2 appears to be an essential adipose tissue-specific enhancer of adipocyte development that is produced throughout adipogenesis [66]. Ectopic overexpression of PPARg2 alone is sufficient to induce pluripotent stem cells to differentiate into adipocytes [67], making PPARg2 both necessary and sufficient for adipogenesis. We used antibodies targeting the distinct amino terminus of PPARg2 to identify subsets of adipocyte nuclei from non-adipocyte nuclei prepared from adipose tissue.

**Fig 3:**
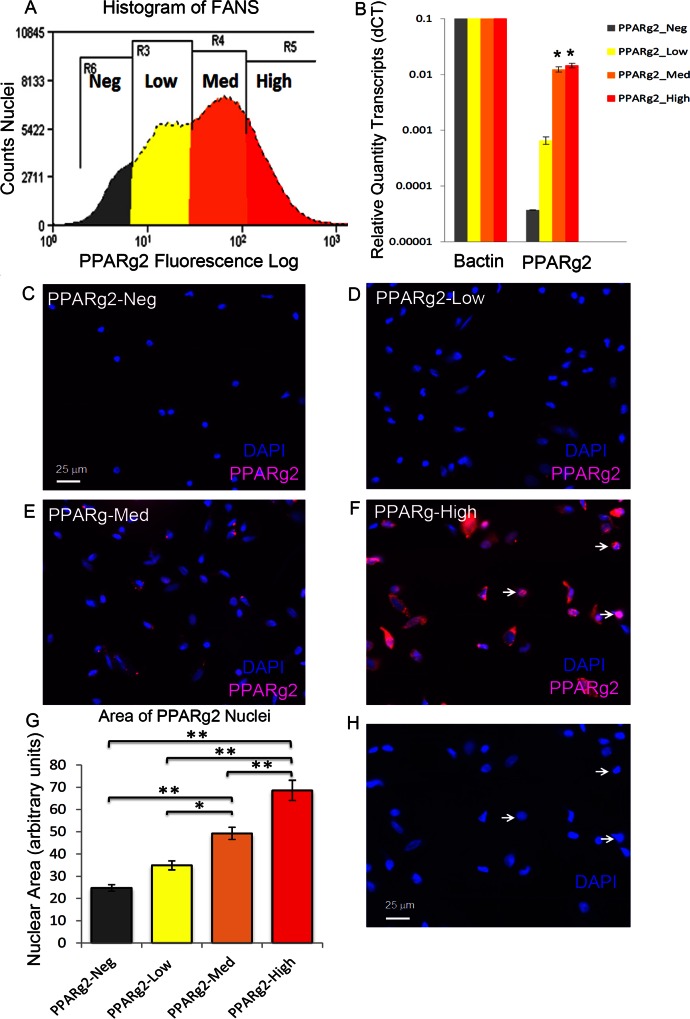
Fluorescence activated nuclear sorting (FANS) of three classes of visceral adipose tissue cellular nuclei immuno-stained for PPARg2. **A.** Histogram of PPARg2-Neg, -Low, -Med. and -High stained nuclei from sorting experiment (log scale) gated for DNA content (DAPI subset, S1B Fig) and forward and side light scattering (S1A Fig). **B**. *PPARg2* transcript levels were assayed among the four fractions of nuclei by qRT-PCR. **C, D, E, F.** Merged immunofluorescence microscope images of four isolated fractions of nuclei without re-staining. **G.** Comparison of the average nuclear area for the four fractions from one experiment (N = 100). There were statistically significant differences between all of the fractions of nuclear areas except for between PPARg2-Low and PPARg2-Neg fractions. **H.** PPARg2-High nuclei from image H showing the DAPI staining alone to reveal decondensed nuclei, however, some strongly stained PPARg2-High nuclei are small reflecting some heterogeneity in their morphology, as indicated by white arrows. For antibodies see S1 Table. A p value of P<0.01 is denoted by an asterisk (*) and a p value of P<0.001 is denoted by a double asterisk (**).

A small subset of VAT nuclei was strongly stained with PPARg2 antibodies and there were also populations of intermediately stained and unstained nuclei as well (S3B Fig). Examination of the highly stained PPARg2-High nuclei for DAPI staining morphology revealed that many were larger and more decondensed with diameters exceeding twice that of the smallest PPARg2 negative nuclei (S3C Fig). We previously defined decondensed to mean that the nuclei had a larger DAPI staining area with less intense staining per unit area as compared to the intensely staining normal-sized 2C nuclei [28]. FNC showed that there was an approximately 100-fold dynamic range in the PPARg2 immuno-fluorescence staining intensity among VAT nuclei (Fig 3) above the background staining observed with secondary antibody alone (S1D Fig).

VAT nuclei were subjected to FANS based on the levels of PPARg2 immuno-staining (Fig 3). Four populations of nuclei were sorted (PPARg2-Neg, -Low, -Med and -High) based on PPARg2 staining, each having an approximately 5-fold increase in PPARg2 immuno-staining intensity above background staining (Fig 3A). The low background staining from the secondary antibody alone was used to define the PPARg2-Neg class of nuclei (S1D Fig). In repeated FANS experiments, we gated to collect approximately 20%, 40%, 30%, and 10% of the nuclei in each category, -High, -Med, -Low, and -Neg, respectively. The sorted nuclear fractions were re-photographed without re-staining (Fig 3C–3F). Besides the obvious difference in PPARg2 staining intensities, there is variation in nuclear morphologies. The PPRG2-Neg nuclei are mostly spherical, while the PPARg2-Med and -Low fractions contained many ovoid- and spindle-shaped nuclei typical of nuclei in the thin cytoplasmic layer surrounding the lipid body in mature adipocytes. The PPARg2-High nuclei are generally oval or round, and were predominantly larger and more decondensed relative to the other populations. However, it is worth noting that some strongly stained PPARg2-High nuclei are small, reflecting some heterogeneity in morphology (White Arrows, Fig 3F and 3H). The PPARg2-Neg nuclei in Fig 3C had a diameter of approximately 6.5 μm, typical of 2C mammalian nuclei [26]. By comparing PPARg2-Neg nuclei (Fig 3C) to PPARg2-High nuclei (Fig 3H), where the PPARg2-High nuclei are viewed for DAPI staining alone, the larger diameters of these nuclei were easily seen. When the average two-dimensional cross-sectional area of the original images of DAPI stained PPARg2-Neg nuclei were set to 1.0, the nuclear areas of the PPARg2-High, -Med, and -Low populations were 2.8-, 2.0-, and 1.6-fold larger, respectively (Fig 3G). There were statistically significant differences among pairwise comparisons of nuclear areas of all the fractions (* p<0.01; ** p<0.001) except for the comparison of the PPARg2-Low and PPARg2-Neg fractions. Hence, the nuclear volumes of adipocyte nuclei may be computed to vary over a 20-fold range. PPARg2 transcript levels were assayed among the four fractions of nuclei by qRT-PCR. *PPARg2* RNA was expressed at significantly higher (~200-fold higher) levels in PPARg2-High and PPARg2-Med classes of nuclei, relative to PPARg2-Neg fraction (* p<0.01, Fig 3B). These data are in reasonable agreement with the approximately 25- to 125-fold higher levels of PPARg2 protein immunofluorescence detected in these two fractions relative to the PPARg-Neg fractions used as the basis for FANS (Fig 3A).

### Transcript profile of sorted visceral adipocyte nuclei Approach

Considering that neither FANS or PPARg2 staining has been implemented to separate and characterize adipose tissue nuclei previously, we profiled the relative levels of a few sets of transcripts, which were potentially informative as to the phenotypes of these four classes of nuclei.

#### Transcripts encoding cell-type-specific markers

**Fig 4:**
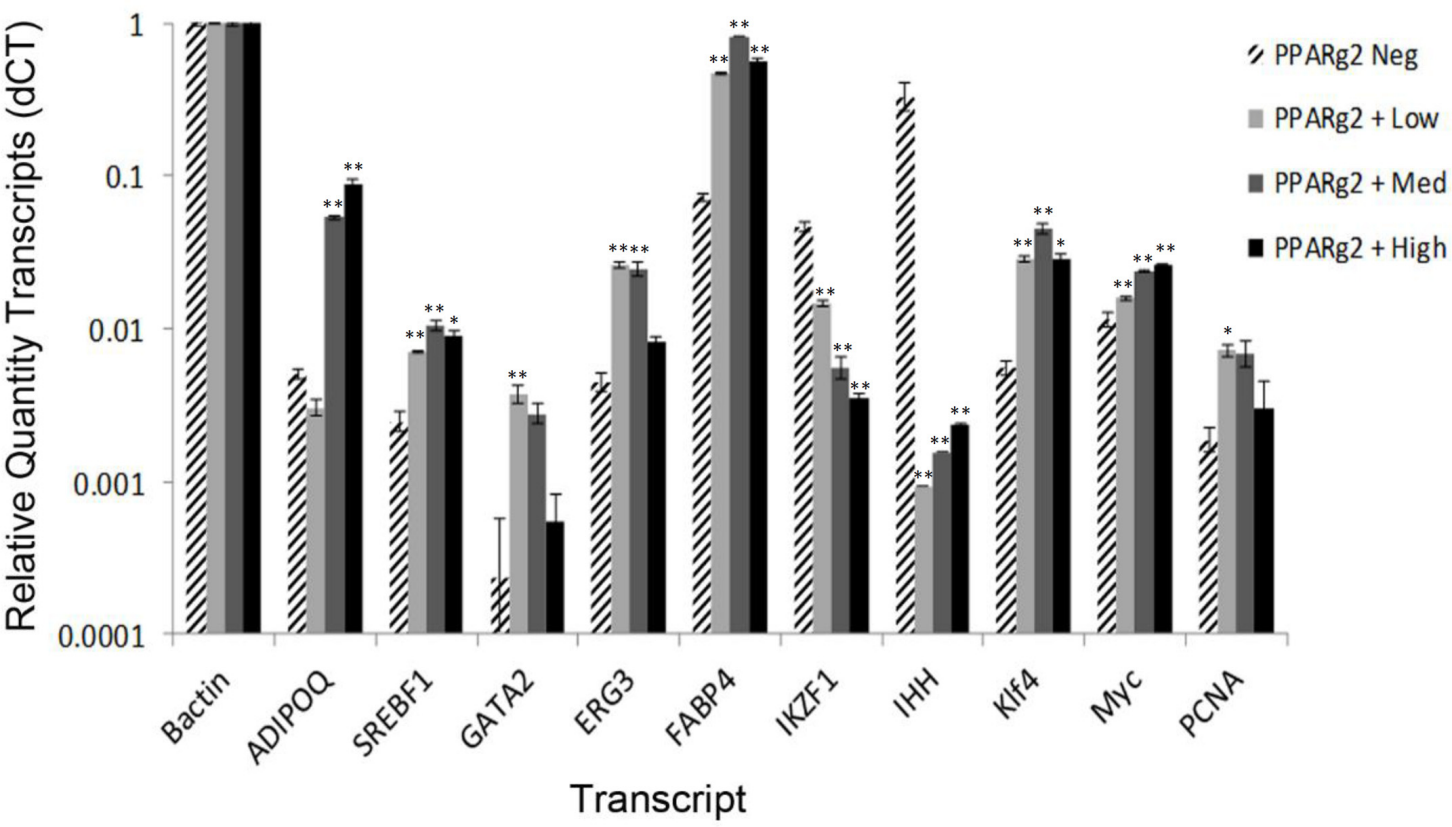
Transcript profiles for factors associated with cell type, pluripotency, and the cell cycle. Relative quantities (RQ) of marker transcripts among the four classes of VAT cell nuclei isolated by FANS were determined by qRT-PCR. The RQ of transcript level was calculated based on the dCT method including the standard deviation from the mean. Beta-actin was used as the endogenous control. Assays were run in triplicate and standard errors are shown (See Materials and Methods). The oligonucleotide primers used and marker genes assayed are described in S2 and S4 Tables, respectively. A p value of P<0.01 is denoted by asterisk (*) and a p value of P<0.001 is denoted by double asterisk (**).

The four classes of adipose tissue nuclei were assayed for the relative quantity (RQ) of transcripts encoding proteins that were reasonably specific markers of adipocytes, endothelial cells, and leukocytes and their potential for cell cycle activity. Among the four fractions, *beta-actin (ACTB)* mRNA was determined to be an equivalently expressed endogenous control relative to total cDNA input. Therefore, the expression levels of various marker transcripts were compared to actin set to 1 (Materials and Methods). For most of the cell type markers examined, significant differences in transcript expression levels were found that distinguished the various PPARg2-Pos classes of nuclei (PPARg2-High, -Med, -Low) from PPARg2-Neg class of nuclei (* P<0.01, ** P<0.001, Fig 4). Adipocyte specific transcripts *ADIPOQ*, *SREBF1*, and *FABP4* were approximately 4- to 20-fold more highly expressed in most of the PPARg2-Pos classes of nuclei, relative to PPARg2-Neg fraction, as shown in Fig 4. The leukocyte and progenitor cell markers *IKZF1* and *IHH* were estimated to be 10- to 100-fold more highly expressed in the PPARg2-Neg fraction of nuclei than in the PPARg2-Pos fractions (Fig 4). IHH, a suppressor of adipocyte development, was extremely highly expressed in PPARg2-Neg nuclei. Thus, the PPARg2-Neg fraction appears enriched for some nuclei from progenitor cell types not committed to adipocyte development. Interestingly, transcripts for the transcription factor GATA2, which promotes the differentiation of MSCs into adipocytes [68], and ERG3, a sterol C5-desaturase involved in cholesterol biosynthesis [69], were more highly expressed in the PPARg2-Low and -Med classes of nuclei than either the PPARg2-Neg or PPARg2-High nuclei. Perhaps, the cells from which PPARg2-High nuclei were derived have finished their development from MSCs and are no longer synthesizing as much lipids. In summary, the PPARg2-Pos nuclei appear to be derived from adipocytes, and the PPARg2-Neg nuclei from non-adipocytes. Summary information and references on the properties of the marker genes assayed is given in S4 Table.

#### Transcripts encoding factors involved in multipotency and cell cycle

Highly decondensed nuclei are associated with the elevated expression of genes for multipotency and chromatin remodeling machinery as shown for decondensed neuronal nuclei in the brain [28] and a differentiating hematopoietic stem cell line [70]. Three markers of cellular multipotency, proliferation, and cell cycle activity were examined in the four nuclear fractions (*KLF4*, *MYC*, and *PCNA* as defined in S4 Table). They were 2- to 6-fold more highly expressed in most of the PPARg2-Pos adipocyte fractions relative to the PPARg2-Neg non-adipocyte fraction of nuclei. Thus, although there was significant variation in the expression of these markers, they were in general more highly expressed in PPARg2-Pos nuclei.

#### Transcripts encoding chromatin-remodeling proteins

**Fig 5:**
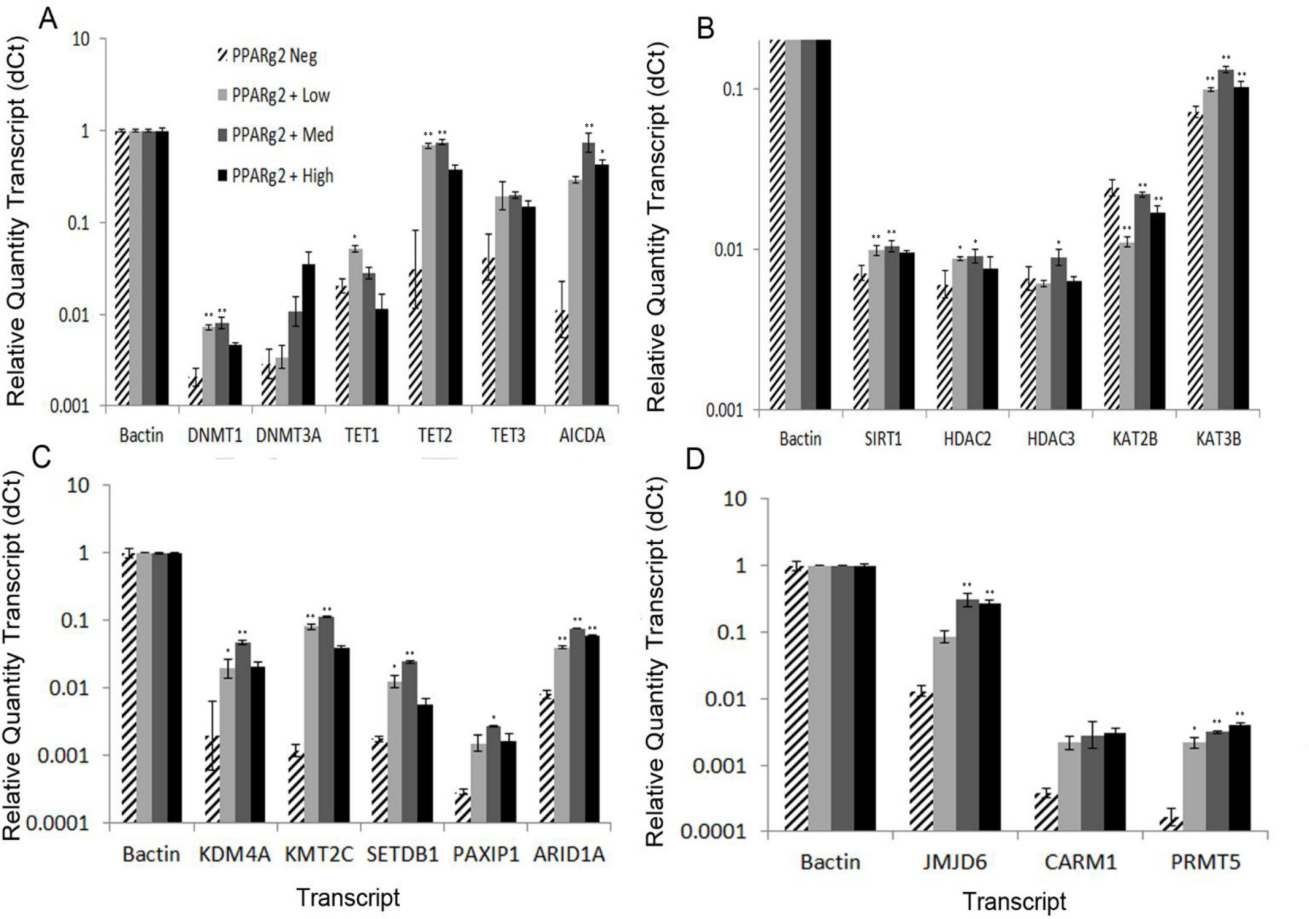
Transcript profiles for factors controlling chromatin modification. Relative quantities of marker transcripts among the four classes of VAT cell nuclei isolated by FANS were determined by qRT-PCR. **A.** Factors controlling the levels of 5-methylcytosine modification of DNA *(DNA cytosine methyltransferases DNMT1* and *DNMT3A*, *methylcytosine dioxygenases (TET1*, *TET2*, *TET3)*, and *activation-induced cytidine deaminase (AICDA))*. **B.** Factors controlling acetylation of nucleosomal histones *(deacetylases Sirt1*, *HDAC2*, and *HDAC3* and *histone lysine transacetylases KAT2B and KAT3B)*. **C.** Factors involved in histone lysine methylation (*ARID1A/BAF250*, *MLL3*, *and PAXIP1/PTIP* impact histone H3 methylation at lysine 4, and *SETDB1* and *KDM4A* impact histone H3 methylation at lysine 9). **D**. Factors involved in histone arginine methylation (*JMJD6* is histone arginine demethylase and *CARM1/PRMT4* and *PRMT5* are arginine methyltransferases). See legend to Fig 4 for details of the RQ calculation. A p value of P<0.01 is denoted by asterisk (*) and a p value of P<0.001 is denoted by double asterisk (**).

To begin testing the first part of our hypothesis, adipose tissue contains epigenetically distinct subpopulations of adipocytes, we analyzed transcripts encoding factors responsible for programming a range of chromatin modifications (Fig 5). Highly decondensed nuclei are associated with an elevated transcription level of chromatin remodeling machinery and genes of multipotency and in the brain with elevated expression of markers for learning and memory [28]. Therefore, although the cellular memory of adipocytes might be biochemically quite different from that of neurons, subsets of adipocyte nuclei still might have different capacities to record cellular memories, and hence, be distinctly potentiated to respond to their tissue environment. We performed qRT-PCR assays on transcripts from 19 genes encoding chromatin-remodeling factors, which are broken into four sets. First we assayed factors involved in DNA cytosine modification including two DNA cytosine methyltransferases, DNMT1 and DNMT3A, which catalyze the synthesis of DNA 5mC and three ten-eleven translocation methylcytosine dioxygenases, TET1, TET2, and TET3, which catalyze the oxidation of 5mC to 5hmC (Fig 5A). TETs are the major enzymes controlling the removal and turnover of 5mC [71]. Of these *DNMT1*, *DNMT3A*, *TET1*, and *TET3* transcripts were 3-, 6-, 1.5-, and 4-fold more highly expressed in PPARg2-Pos (PPARg2-High, -Low and -Med) adipocyte factions, respectively, relative to the non-adipocyte faction. *TET2* and *AICDA* were 19- and 40-fold higher more highly expressed in the PPARg2-Pos fraction.

Second, we considered histone side chain acetylation (Fig 5B), but the differences among nuclear fractions appeared less dynamic. Transcripts for two histone lysine acetyltransferases, *KAT2B* and *KAT3B*, and three histone deacetylases SIRT1, *HDAC2*, and *HDAC3*, were assayed. Only the transcripts encoding *HDAC2*, *KAT3B*, and *SIRT1* deacetylase were notably more highly expressed (e.g., ~2-fold) in PPARg2-Pos nuclei than negative nuclei, although there were also quantitative differences among the nuclear fractions for *KAT2B* and *HDAC3*.

Third, transcript levels for four factors involved in nucleosomal histone side chain methylation (S4 Table) were quantified (Fig 5C). This included lysine-specific demethylase *KDM4A*, lysine specific methyltransferase *KMT2C*, histone H3K9 methyltransferase *SETDB1*, a cofactor that promotes histone lysine methylation *PAXIP1*, and Swi/Snf related helicase ATPase *ARID1A*, which is known to modulate H3K4me1 nucleosomes. Fourth, we examined the protein arginine methyltransferases *CARM1 and PRMT5* and demethylases, including histone arginine demethylase *JMJD6*. The transcripts of these last two classes of genes were 6- to 65-fold more highly expressed in PPARg2-Pos nuclei than PPARg2-Neg nuclei (Fig 5D). Clearly, for the PPARg2-Pos nuclei there were much higher levels of factors involved in histone methylation than factors involved in histone acetylation.

### IFM analysis of chromatin modifications in isolated nuclei

**Fig 6:**
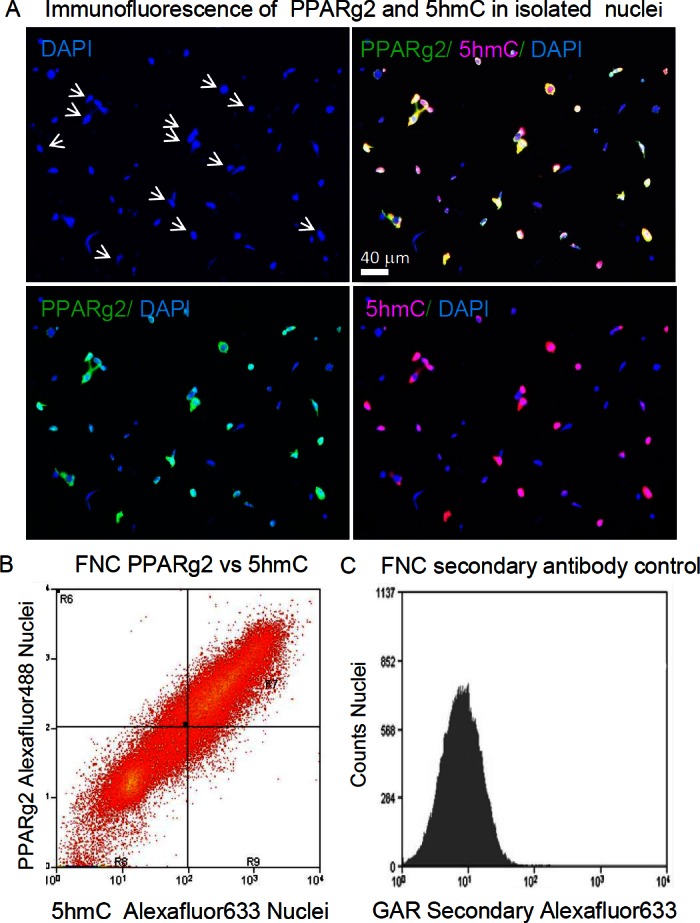
Distribution of 5hmC among adipose tissue nuclei. IFM and FNC were used to examine 5hmC levels among visceral adipose tissue nuclei. **A.** A field of VAT nuclei examined with various combinations of DAPI staining for DNA, and immunostaining with mouse anti-PPARg2 + goat anti-mouse Alexafluor488 and rabbit anti-5hmC + goat anti-rabbit Alexafluor633. White arrows indicate those large, decondensed nuclei that are stained strongly for both 5hmC and PPARg2. **B.** Flow Cytometry of VAT nuclei immunostained as in A. **C.** Goat anti-rabbit secondary antibody used in B shows only modest background staining of nuclei. Nuclei were gated for DAPI (>2C DNA content) and size and shape by light scattering as in S1 Fig. For antibodies see S1 Table.

Because strong differential expression of transcripts encoding proteins involved in DNA and histone methylation was observed, one DNA and two histone modification products were assayed. First, the TET catalyzed oxidation of 5mC to 5hmC and 5hmC levels themselves are often dynamically regulated in the development of stem cells, germ cells, T cells, and neurons [38,72,73]. Therefore, we performed a semi-quantitative IFM of 5hmC levels [74,75] among the various fractions of VAT nuclei. Preliminary experiments showed 5hmC was concentrated in large decondensed nuclei (Fig 6). When we examined the co-distribution of PPARg2 protein with 5hmC, nearly all nuclei staining most strongly for PPARg2 also stained most strongly for 5hmC (White Arrows, Fig 6A). The coordinate expression of PPARg2 and 5hmC was examined further by FNC (Fig 6B and 6C). The cytometer resolved a wide, nearly 100-fold, range of positive staining for both markers. Nuclei stained with the secondary antibody alone used to detect 5hmC helped define background fluorescence. As PPARg2 also defined nuclear size (Fig 3H), perhaps this correlation of 5hmC with PPARg2 levels should not be surprising, considering the evidence that 5hmC marks decondensed euchromatin [76,77].

### Levels and gene-region distribution of 5hmC

In view of the large differences in expression levels of factors controlling DNA cytosine methylation and turnover via 5hmC (Figs 1 and 5A) and the association of 5hmC with decondensed highly active chromatin, it seemed reasonable to consider that 5hmC levels might vary widely among the fractionated VAT nuclei and be essential to their epigenetic programming. We performed TAB-seq to evaluate 5hmC levels in three classes of VAT nuclei isolated by FANS (PPARg2-High, pooled PPARg2-Med and -Low, and PPARg2-Neg). The specificity of TET-enzymes and their cofactors, results in 98% of 5hmC being in the CG dinucleotide context. Therefore, the data on 5hmC levels are reported as a fraction or percent of CG dinucleotides. The percent of 5hmCG ranged from 3.40%, to 3.03% to 2.22% (scaled %5hmCG value is 6.50%, 6.00% and 4.58%, respectively) of CG dinucleotides among these three classes of VAT nuclei (Table 1). The scaled %5hmCG values are the true 5hmCG levels in the genome as scaling corrects for varying degrees of protection rates in the TAB-seq assay. The scaled %5hmCG levels in PPARg2-High and in PPARg2-Med+Low nuclei were significantly higher than that in PPARg2-Neg population (Chi-square test p-value <0.05).

**Table 1:** Percent 5hmCG in three classes of nuclei determined by TAB-seq and averaged across all sequences.

Nuclei	Total 5hmCG	Total CG	% 5hmCG	Scaled % 5hmCG
PPARg2-High	159,774	4,695,778	3.40%	6.50%
PPARg2-Med+Low^a^	117,954	3,886,756	3.03%	6.00%
PPARg2-Neg	88,910	4,004,237	2.22%	4.58%

The percent 5hmCG (% 5hmCG) was calculated for three classes of nuclei by dividing the total 5hmCG assayed by the total CG content. The scaled %5hmCG values were calculated by dividing %5hmCG values by the corresponding pUC19 protection rates. PPARg2-Med+Low^a^: is the pooled PPARg2-Med and PPARg2-Low nuclear populations.

**Fig 7:**
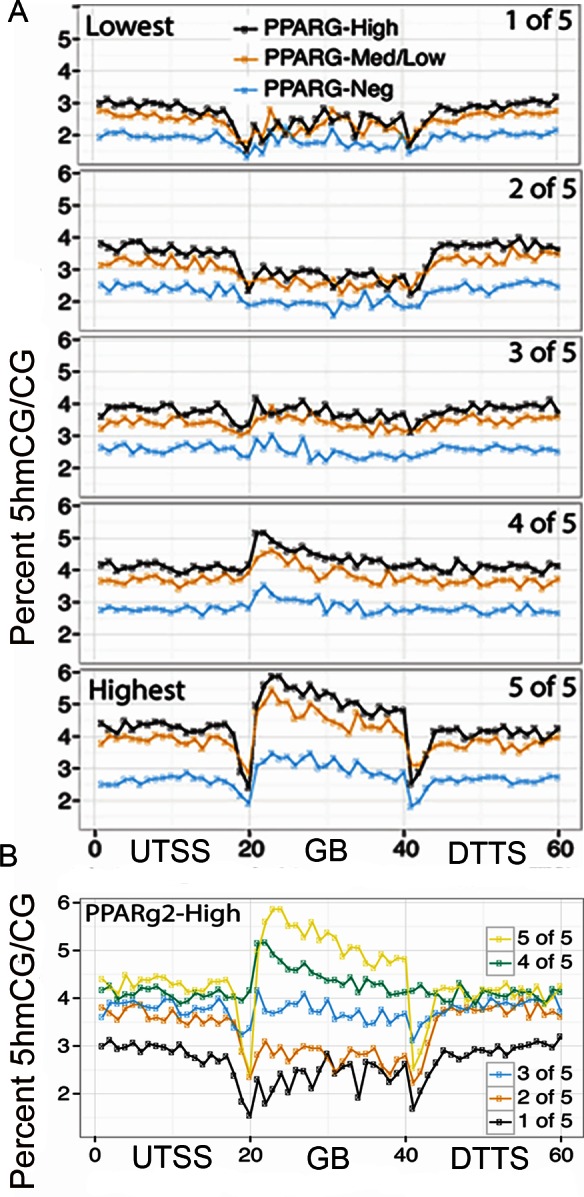
5hmC levels vary dramatically across gene regions based on transcript levels in adipose tissue and the class of VAT nuclei. 25,321 *S*. *scrofa* genes were ranked into five quintiles (5,064 to 5,065 genes in each) based on the levels of adipose tissue transcript expression determined by RNAseq (1st quintile represents the lowest levels of RNA expression, 5th quintile the highest). **A.** The differences in gene region 5hmC levels for PPARg2-High, -Low, and -Neg nuclei are shown for each quintile of expressed genes. PPARg2-Neg nuclei had the lowest 5hmC levels in each quintile, while PPARg2-High nuclei had slightly higher 5hmC levels than PPARg2-Low nuclei. **B.** 5hmC levels are shown for all 5 quintiles for the PPARg2-High, PPARg2-Med+Low, and PPARg2-Neg nuclei. **A & B.** 5hmC levels were lower in the gene body relative to the flanking gene regions for the two lowest quintile expression groups of transcripts, whereas for the 3 highest transcript expression groups the pattern was reversed with 5hmC levels being the highest in the gene body. Gene regions were divided into three parts: 100 kb upstream of the transcription start site (UTSS), gene body (GB, TSS to transcription stop site (TTS)), and 100 kb downstream of the TTS (DTTS).

We compared the gene-region distribution of 5hmCs among the three classes of VAT nuclei for 25,321 genes divided into quintiles based on RNA-seq expression data in adipose tissue [56]. Gene regions were divided into three parts: 100 kb upstream of the transcription start site (UTSS), 100 kb downstream of the transcription stop site (DTTS), and the gene body (GB) extending from TSS to TTS. 5hmC data were estimated from gene sequences divided into 20 equal bins for each region and the fraction or percent 5hmCG per CG dinucleotide was calculated. For the highest quintile of expressed genes (5 of 5, Fig 7A), the pattern of 5hmCG distribution begins with a deep valley in 5hmCG levels at the TSS, rises to a high broad plateau across the gene body, and ends with another steep valley of 5hmCG at the TTS. Across all gene regions, 5hmCG levels were the highest for PPARg2-High nuclei and lowest for PPARg2-Neg nuclei, although the pooled PPARg2-Med and -Low nuclear populations contained only slightly lower levels than that of the -High population. For the 3^rd^ and 4^th^ quintile expression gene groups the distribution of 5hmCG was relatively indistinct, although there was small peak in 5hmCG levels right after the TSS. Surprisingly, the 5hmC levels drop across the gene body for the lowest two quintiles (1^st^, 2^nd^) for all three classes of cellular nuclei. As far as we are aware a gene region drop in 5hmC has not been reported for any other gene set. Fig 7B compares the wide range in 5hmC levels and differences in patterns of 5hmC distribution among the five quintiles for the PPARg2-High nuclei.

## Discussion

### Manipulating cellular nuclei from adipose tissue

Considerable progress was made in simplifying the isolation of total cellular nuclei from within adipose tissue as a new tool for cell-type-specific analyses. Adipocyte, endothelial cell, and lymphoid cell nuclei were easily isolated from VAT using only slight modifications to an existing rapid protocol for isolating brain cell nuclei. The method required only bench top centrifugation through a sucrose cushion and two filtration steps and did not require ultracentrifugation as in earlier established methods [78]. Nuclei had sufficient purity from cytoplasmic debris to greatly simplify analysis by IFM, FNC, and FANS. The PPARg2 isoform of PPARg was adipocyte cell-type-specific and expressed strongly enough to identify adipocyte nuclei, while the PPARg1 isoform was not. The relative cell-type purity of the PPARg2-Pos adipocyte nuclear populations was validated by the quantitative assessment of cell-type-specific transcripts. Hence, PPARg2 appears to be a reasonable choice as a pan-adipocyte marker, although PPARg2 labeled nuclei from other fat deposits and from other species will have to be examined.

Quantitative assessment of nuclear transcripts in the different sorted subpopulations of VAT nuclei revealed differential expression of some important markers over more than two orders of magnitude, providing significant resolution for expression studies. This result agrees with previous RNA expression studies on isolated sub-populations of cellular nuclei from plant roots and mouse brain [22,26,28,38]. We showed the utility of using PPARg2 as an adipocyte marker for IFM and FNC. More particularly, the three subsets of adipocyte nuclei that differed in ~5-fold increments in the levels of PPARg2 expression, displayed significant differences in the expression levels of some markers, although most epigenetic markers of adipocyte identity and cell cycle activity simply distinguished adipocyte PPARg2-Pos from PPARg2-Neg nuclei.

### Differential programming of distinct adipocyte populations

Our data give strong initial experimental support for the first part of our working hypothesis by showing that adipose tissue contains subsets of adipocytes that are epigenetically distinct. The four populations of VAT nuclei sorted based on PPARg2 protein levels differed significantly in the expression of transcripts encoding factors involved in chromatin modification and/or adipogenesis. Nineteen of the twenty-two transcripts associated with epigenetic control, pluripotency, and/or the cell cycle that were assayed showed 2- to 100-fold differences in their levels of expression among the four populations. Transcript levels were particularly distinct between PPARg2-Pos adipocyte nuclei and PPARg2-Neg non-adipocyte nuclei. IHH is the only hedgehog morphogen known to be expressed in preadipocytes, where it inhibits adipogenesis and promotes chondrocyte differentiation, proliferation, and maturation. Transcripts for IHH were far more highly expressed in non-adipocyte PPARg2-Neg nuclei than in adipocyte nuclei. IHH should not be expressed in maturing or mature adipocytes, and hence, served to confirm the identity of sorted nuclear populations of preadipocytes. We found 2- to 50-fold differences in the expression of genes specifically associated with different stages of adipogenesis including *ADIPOQ*, *SREBF1*, *GATA2*, *ERG3*, and *FABP4*. Three markers of pluripotency and cell cycle potential, *KLF4*, *MYC*, and *PCNA*, were 2- to 6-fold more highly expressed in adipocyte populations, suggesting perhaps there is reasonable developmental potential among diverse classes of adipocytes.

The discussion of chromatin remodeling factors has been divided into two parts concerning the formation and removal of (1) histone modifications and (2) DNA cytosine methylation.

#### Histone PTMs

We analyzed transcripts for 13 enzymes or protein subunits of enzyme complexes involved in making or removing histone side chain PTMs and directly assayed two PTMs directly on nucleosomes in nuclei. Many of these PTMs have been correlated with transcription in adipocytes and with adipogenesis, if not with the suggestion that they are playing a direct causal role in cellular differentiation (S4 Table).

#### Histone lysine acetylation

The forced down regulation of histone deacetylases promotes adipogenesis [79,80], however, only barely detectable changes are observed in global histone acetylation PTMs during adipogenesis of 3T3-L1 cells [81]. In agreement with these latter results, we found only small to insignificant differences in transcript levels for representative deacetylases SIRT1, HDAC2, and HDAC3 and histone lysine transacetylases KAT2B and KAT3B among the three classes of adipocytes (Fig 5B). Instead of examining the expression of histone transacetylase and deacetylase transcripts, a direct analysis of various histones modified by acetylation within populations of PPARg2-Pos adipocyte nuclei by FNC might be more informative.

#### Histone lysine methylation

Transcripts for five of the chromatin remodelers assayed (ARID1A/BAF250, MLL3, PTIP, SETDB1, KDM4A) impact histone H3 methylation at lysine 4 and/or 9. They shared an interesting common profile of differential transcript expression, being much more highly expressed in PPARg2-Pos adipocyte nuclei than non-adipocyte nuclei, and were the most highly expressed in PPARg2-Med nuclei. The differential methylation of nucleosomal histone H3 appears to play major roles in regulating adipogenesis [82,83]. The nucleosomes associated with CEBPA, CEBPB, PPARg2 and aP2 gene sequences show significant increases in the levels of H3K4me1 in the later stages of 3T3-L1 preadipocyte differentiation into mature adipocytes [81]. H3K4me1 is found in the enhancers of genes potentiated for expression [84]. By contrast, nucleosomal H3K9me1 is found in the promoters of actively expressed genes [84]. Conversion to more highly methylated H3K9me2 at the PPARg locus is associated with repressed adipogenesis [30]. Surprisingly, these two PTMs are primarily associated with subsets of expressed genes after hyperglycemic 3T3 cells are stimulated with insulin [85]. Further confusing their potential stimulatory or inhibitory role in adipogenesis, both PTMs have been found to be associated with silenced genes in euchromatin [86–88]. Perhaps the relevant association of H3K4me1 and H3K9me1 is with decondensed chromatin, which appears proportional to PPARg2 expression in adipocytes.

This discussion will focus briefly on the observed differential expression of three factors controlling H3K4me1 and H4K9me1 levels among classes of VAT nuclei: ARID1A, KMT2C, and PAXIP1 [85]. ARID1A is the large Swi/Snf ATPase subunit defining many BAF remodeling complexes including complexes that methylate H3K4 to H3K4me1 [89,90]. ARID1A complexes regulate pluripotency genes and are essential to the conversion of ES cells into adipocytes [91]. KMT2C is a histone lysine methyltransferase that methylates H3K4 to H3K4me1 and me2 [92,93] and is physically associated with the lineage-specific enhancers and cell-type-specific factors including *PPARg* and *FABP4* [92]. *KMT2C* mutant mice have less white fat and are defective in adipogenesis [94]. PAXIP1 binds to histone 3 lysine 4 methyltransferases to influence the conversion of H3K4me1 to H3K4me3 in nucleosomes associated with the promoter regions of *PPARg* and *CEBPA*. PAXIP1 is essential to their induced expression, and hence, essential to adipogenesis [95], but its activity acts in opposition to ARID1A and KTM2C because it reduces H3K4me1 levels, whereas the latter increase it. Considering that all three factors are essential to adipogenesis and that PPARg is essential to this process, it is not surprising that PPARg2-Pos adipocyte nuclei express these remodelers at much higher levels than non-adipocytes. Because turnover rates for chromatin modifications are a function of their synthesis and decay (i.e., removal) rates [29], the coordinately higher expression of these factors with opposite activities in adipocytes relative to non-adipocytes suggests more rapid turnover rates for H3K4me1.

Next, we consider the two factors regulating the levels of nucleosomal H3K9me1, SETDB1 and KDM4A. SETDB1 is a H3K9 methyltransferase that represses PPARg transactivation via nucleosomal histone methylation at PPARg target genes. SETDB1 methylates H3K9 and H3K9me1 to H3K9me3, a modification associated with transcriptional repression [96]. Conversely, KDM4A is a lysine-specific demethylase that directly demethylates H3K9me3 to H3K9me1/2 [97,98]. Very early in the differentiation of 3T3-L1 preadipocytes levels of the repressive H3K9me3 mark increase 2- to 3-fold, “licensing” preadiopcytes to differentiate into mature adipocytes [44]. KDM4A is essential to recruiting PPARg to the many target genes expressed during adipocyte development [99]. It is reasonable to consider that KDM4A-catalyzed conversion of H3K9me3 to H3K9me1 directs adipogenesis to proceed. The higher levels of these two opposing activities in PPARg2-Pos adipocyte nuclei may result in an increased turnover rate for H3K9 related methylation.

#### Histone arginine methylation

We examined the transcript levels of two histone arginine methyltransferases, CARM1 (PRMT4) and PRMT5, and one arginine demethylase, JMJD6 [100,101]. We showed that CARM1, PRMT5, and JMJD6 transcripts were 7-, 18-, and 17-fold more highly expressed in PPARg2-Pos adipocyte nuclei than PPARg2-Neg non-adipocyte nuclei, respectively. Among their multiple activities on protein substrates, CARM1 and PRMT5 are both capable of generating monomethylarginine (MMA) and then, respectively, they may synthesize asymmetric dimethyl arginine (ADMA) and symmetric dimethyl arginine (SDMA). JMJD6 activity can catalyze the demethylation of MMA, ADMA, and SDMA residues in some protein contexts. All three enzymes appear essential to adipogenesis, based for example, on the evidence that small RNA silencing of CARM1 [102] or PRMT5[103] or JMJD6 [104] each results a an approximate 90% reduction of in situ adipogenesis starting with embryonic stem cells or preadipocytes. By contrast, another member of the family of 9 PRMTs, PRMT7, is not needed for adipogenesis, emphasizing the specificity of CARM1/PRMT4 and PRMT5 [105]. The higher levels of these two opposing activities (i.e., CARM1 and PRMT5 methyltransferases vs JMJD6 demethylase) in PPARg2-Pos adipocyte nuclei suggest an increased turnover rate for the methylation of some arginine residues in adipocytes relative to other adipose tissue cell types.

### TET expression and 5hmC

We began with a preliminary examination of the levels TET expression and 5hmC levels in nuclei fractionated based on PPARg2 levels. PPARg2 is the major transcription factor driving adipogenesis and lipid synthesis in mature adipocytes. It acts via its binding to PPARg enhancers (PPAREs). Using IFM we found that all three TETs proteins (TET1, 2, 3) were present at significantly higher levels in PPARg2-High adipocyte nuclei than in most adipocyte nuclei staining moderately for PPARg2 or PPARg2-Neg non-adipocytes. Based on cytometry, total 5hmC appeared proportional to PPARg2 levels in nuclei. However, qRT-PCR data only showed moderate differences in TET RNA expression among fractionated nuclei and only TET2 and TET3 levels were significantly higher in PPARg2-High nuclei. DNMT1 transcript levels were relatively higher in all three classes of PPARg2-Pos nuclei compared to PPARg2-Neg, but the lowest level among these was seen in the PPARg2-High samples, perhaps reflecting the decline in DNMT1 reported for fully mature adipocytes [45].

Similarly, we found exceptionally high levels of 5hmC in PPARg2-High nuclei relative to the balance of VAT nuclei by IFM and observed their coordinate expression over more than an order of magnitude by FNC. Our TAB-seq data confirmed that PPARg2-Pos nuclei had the highest levels of 5hmC, significantly higher than PPARg2-Neg nuclei. The 2.2 to 3.4% 5hmCG per CG dinucleotides observed in VAT nuclei were low as compared to the estimated 13% 5hmCG in adult brain where 5hmC levels are the highest [106], but this represents an intermediate level among estimates for many other tissue types [107,108]. Yet, by TAB-seq there were only slightly higher levels of 5hmC in the PPARg2-High nuclei compared to the balance of PPARg2-Low/Med nuclei. The TAB-seq method undoubtedly provides one of the most quantitative and unbiased assessments of the relative levels of 5hmC. Confirmation of the absolute quantitative levels awaits analysis by a method such as LC-MS [107,109].

There are a few straightforward explanations for these differences among measurements made by qRT-PCR for TET RNAs, immuno-detection of 5hmC, and TAB-seq analysis of 5hmC. First, differential stability of TET RNAs and proteins might favor the accumulation of TET proteins in the PPARg2-High subset of cells, while TET RNA levels declined. Second, 5hmC is most concentrated in euchromatin in regions with decondensed structure [76,77,110]. We observed that PPARg2-High nuclei were extremely large and decondensed and nuclear size appears to be proportional to the levels of both PPARg2 and 5hmC detected with antibodies. Both IFM and nuclear cytometry (FNC, FANS) showed a wide dynamic range for the immunological detection of PPARg2 protein and 5hmC. Perhaps a decondensed chromatin structure provides disproportionate access to immune reagents amplifying the difference in immunochemical staining among the nuclear fractions relative to condensed chromatin is other nuclei blocking access. While this potential artifact would prevent precise quantitative interpretation of our immunochemical data, it may have contributed to the wide dynamic ranges of PPARg2 and 5hmC staining observed and aided in separating classes of PPARg2 stained nuclei by FANS.

### Gene-region distribution of 5hmC

TAB-seq analysis of three classes of VAT nuclei showed that 5hmC was concentrated in the gene bodies of the highest quintile of expressed genes, above the levels in flanking regions, and was much higher for PPARg2-Pos nuclei than PPARg2-Neg. Perhaps this relationship between PPARg2 and 5hmC may not be too surprising, considering the recent evidence that PPARg bound to PPAREs attracts TET enzymes and this results in the chromatin-localized conversion of 5mC to 5hmC [42]. 5hmC levels increase during the development of 3T3-L1 preadipocytes into adipocytes. Small RNA silencing of any one or all three TETs prevents this part of the increase, strongly supporting the view that all three contribute to 5hmC levels in adipose tissue [42]. Perhaps 5hmCGs program cellular memory in adipose tissues, tagging sites for demethylation or remethylation at a later time and creating a poised or potentiated state as suggested for the development of neurons in the brain and for embryonic stem cells [37,38,111]. Constitutive CTCF enhancers that are active throughout adipogenesis and PPAREs that become active during adipogenesis are often concentrated in CG rich regions. During adipogenesis, PPARg binding is associated with a dramatic decrease in 5mC levels and an increase in 5hmC levels at both constitutive enhancers and activated PPAREs. Changes in the methylation state of the CTCF and PPARg enhancers activates adjacent gene expression, with notable increases in expression of genes involved in glucose signaling and lipid metabolism [60]. Note that reduced 5mC at these enhancers is in contrast to increases in 5mC levels at enhancers in the C/EBP alpha promoter reported previously [44]. Because the standard whole genome bisulfite sequencing technology to determine 5mC does not distinguish between 5mC and 5hmC, it is reasonable to consider that some of the reported increases in 5mC included increases in promoter and gene region 5hmC. By contrast, simply lowering 5mC levels via treatment with 5-azaC down regulates PPARg and halts adipogenesis of 3T3-L1 cells. One explanation for this complexity is that hydroxymethylation of these CG-rich enhancer regions is required for their subsequent activation, suggesting a possible cause-and-effect direct relationship with 5hmC acting at a high level. TET2 protein does interact with both transcription factors, PPARg and CTCF, to promote DNA hydroxymethylation of 5mCGs at their associated enhancers, CCCTC-related sequences and PPAREs, respectively. Hence, TET activity appears to drive increases in constitutive and adipogenic gene expression during the development of 3T3-L1 preadipocytes into mature, lipid-rich adipocytes [40,41]. The likelihood of a specific relationship between PPARg and CTCF is further evidenced by that fact that during adipogenesis, CTCF binds disproportionately to enhancer sites that are near PPAREs and at most genes induced by PPARg [40]. It has been suggested that 3-dimensional chromatin loops bring these two enhancers into proximity to promote coordinated activity [41]. The resulting specific relationship of PPARg, TET activity, and 5hmC levels in adipocytes may also help explain the lower than average levels of 5hmC we observed in the gene bodies of the lowest quintile of expressed genes. This would occur if PPARg-associated TET activity further oxidizes 5hmCG to 5fCG and 5caCG, which would not only lower 5hmC, but could lead to higher levels of 5mCGs and gene silencing. Future studies will explore the more complex examination of 5hmC levels surrounding these and other enhancers.

### A model for 5hmC activity

Considering our results in the light of other recent publications on 5hmC in the brain suggests a model in which 5hmC enriched open chromatin in adipose tissue-specific gene regions enables appropriate patterns of adipocyte gene regulation. 5hmC levels in neurons are said to “potentiate” changes in gene expression and to prepare for rapid “on demand gene regulation,” but are also proportional to steady state transcript levels [38,43]. Although, cause and effect are not yet well defined in adipose tissue, in the brain increases in a genes 5hmC level are often a prelude to changes in gene expression. Further, the loss of normal TET function causes aberrant gene expression in a number of tissues and organs. Recall that the levels of 5hmC observed in adipocytes are several fold lower than in brain, but still correlated positively with gene expression level. Perhaps the levels of gene region 5hmC and the associated open chromatin environment potentiate the most active gene regions for more rapid changes in transcription, similar to warming up a gasoline engine prior to putting it in gear. In this context, 5hmC levels may act as a throttle regulating the relative transcriptional potential and activity in different regions of chromatin. However, the throttle may be set differently in different tissues, such that the idling speed is different. By this amendment to the model the range of 5hmC-determined idling speeds would be broad among cell types and gene sets in adipose tissue, but still lower than in the brain, reflecting a lower rate of chromatin turnover and a slower rate of cellular memory formation in response to environmental influences relative to neurons. By measuring the relative turnover rates for 5mC and 5hmC in adipocytes and brain, the role of turnover in this model may be tested.

## Conclusions

FNC and FANS offer the technical power to analyze cell-type-specific differences in chromatin structures for less accessible organs and tissues, such as adipose tissue. Cytometry provides vast numerical superiority to the analysis of the distribution of nuclear epitypes such as DNA cytosine or histone modification over any other existing approach. An examination of subpopulations of adipocyte and non-adipocyte nuclei derived from VAT demonstrated there is a wide variation in nuclear morphology and size, chromatin structure, progenitor status, and perhaps the potential to form cellular memories, providing initial support for our hypothesis. The extreme variation in nuclear size among adipocyte nuclei is only partially explained by exceptional transcriptional and epigenetic activities, and warrants further examination, particularly in light of the data from other systems directly correlating large decondensed nuclear morphology with progenitor cell status. The large size of adipocyte nuclei may simply reflect more chromatin remodeling machinery and higher rates of chromatin remodeling, independent of multipotency.

This is the first report of 5hmC levels across gene regions of adipocytes and non-adipocytes isolated from within visceral adipose tissue. We found a wide range in 5hmC levels in gene regions, 5-fold differences among genes and cell types ranked based on their quintile expression level and 4-fold within the quintile expression gene groups. This is twice the difference that has been reported between neurons and non-neurons, even though the total levels of 5hmC are much lower in VAT and adipocytes than they are in the brain and neurons. Some of the greater differences in 5hmC levels we report here may be due to the greater resolution obtained by comparing DNA from more highly enriched cell types. Most unexpected were the extremely low levels of 5hmC observed for weakly expressed genes in their gene bodies, below the levels found in flanking sequence regions. These distinctions suggest important activities for 5hmC in adipose tissue development and/or maintenance, but some of these activities may be different from those in the brain.

A small number of stimulating recent studies demonstrate changes in the genome-wide and gene-specific distribution of 5mC in human adipose tissue in response to obesity, metabolic syndrome, and extended exercise [31,34,112–114]. Our results showing large differences in TET expression and 5hmC levels among classes of adipocytes suggest a complex role for the turnover of modified DNA cytosine in regulating gene expression in adipose tissues. It appears likely that distinct subpopulations of adipocyte nuclei within adipose tissue may be programmed with their own cytosine modification epitype. Each subset may respond differently to stresses in their tissue environment and contribute in different ways to metabolic health. A continued examination of subpopulations of adipose tissue nuclei should greatly improve the statistical significance of epitype data from VAT and should more accurately report epigenome-induced risk of disease.

## Supporting Information

S1 Fig Additional information about *ss*VAT Nuclei sorted by FANS based on PPARg2 staining. Labeled nuclei were gated for size and shape (**A**) and DNA content (**B**) to reduce the number of contaminating particles sorted. There was no significant population of doublet nuclei was detected based on side scatter band width (**C**). **D.** Background fluorescence of PE-conjugated goat anti-rabbit secondary antibody (blue) used to label rabbit anti-PPARg2 for FANS (pink) as shown in detail in Fig 3. The low background staining levels from the secondary antibody were used to define the PPARg2-Neg class of nuclei.
(TIF)

S2 Fig 5hmC metagene plots for six groups of genes from published dataset (Lister et al., 2013) with different coverage. Due to high cost associated with deep coverage of the pig genome using WGBS, we chose an alternative strategy to look at 5hmC metagene plots for hundreds to thousands of genes (groups of genes). To demonstrate that this approach and our levels of coverage (i.e., 0.4X genome equivalents) is robust, we downloaded a published high-coverage 5hmC dataset including all genes in mouse frontal cortex and then subsampled number of reads ranging from 0.2X to 13X genome equivalents of coverage to plot 5hmC distribution for six gene groups. As can be seen, coverage didn’t affect the patterns of metagene plots, even for coverage as low as 0.2X.
(TIF)

S3 Fig *Ss*VAT nuclei staining pattern for PPARg1 and PPARg2. **A**. Nuclei were stained with DAPI for DNA (blue) and PPARg1 (red). Nuclei were stained with monoclonal antibody to PPAR-gamma (Abcam Cat.# ab70405) and then goat anti-mouse IgG conjugated with R-PE (Invitrogen Cat.# P-852) and counter stained with DAPI (blue).**B**. Nuclei were stained with DAPI for DNA (green) and PPARg2 (red). Nuclei were stained with polyclonal antibody to PPAR gamma 2 (Abcam Cat. # ab45036) and then Alexa fluor 633 goat anti-rabbit IgG secondary antibody (Life technologies, A21070) and counter stained with DAPI (green).
(TIF)

S4 Fig The level of PPARg1 was used as an immuno-marker to sort *ss*VAT nuclei. **A.** Histogram of PPARg1-Neg, -Low and -High stained nuclei from sorting experiment (log scale) gated for DNA content and forward and side light scattering (not shown). PPARg1 antibody (Abcam Cat.# ab70405). **B, D, E.** Histograms of three sorted nuclear fractions re-examined by cytometry to confirm their relative PPARg1 staining levels. **E, F, G**. Merged immunofluorescence microscope images of three isolated fractions of nuclei without re-staining with DAPI shown in green and PPARg1 in red.
(TIF)

S5 Fig Nuclei sorted based on levels of immunostained PPARg1 were not well resolved from the nuclei of other cell types based on qRT-PCR analysis of cell type markers. Relative quantities of marker transcripts among the four classes of VAT cell nuclei isolated by FANS were determined by qRT-PCR. Using either *Beta actin* or *RPL13A* as endogenous controls gave similar results. Cell-type-specific markers *ADN*, *SREBF1*, *GATA2*, *ERG3*, *IKAROS*, and *CD31* were examined. The properties of maker genes and the oligonucleotide primers are described in S2 and S4 Tables, respectively.
(TIF)

S1 Table Primary and secondary antibodies used in this paper. Specific information of antibodies used in this paper were listed.
(DOCX)

S2 Table Oligonucleotide primers for qRT-PCR analysis of *Sus scrofa* transcript levels in isolated nuclei. Sense and antisense primer sequences used in this paper were listed.
(DOCX)

S3 Table TAB-seq Analysis Metrics. The genome coverage achieved by TAB-seq was listed in the last column as a fraction of our coverage to *Sus scrofa* reference genome Sscrofa10.2 (GCA_000003025.4).
(DOCX)

S4 Table Summary information and references on the properties of the marker genes assayed in this paper. The symbol, full name and description of the genes assayed in this paper were listed.
(DOCX)

## Abbreviations

ACTB/Bactin: beta actin
ADIPOQ: adiponectin
AICDA: activation-induced cytidine deaminase
ARID1A: SWI-SNF-related AT rich interactive domain 1A
CARM1: coactivator-associated arginine methyltransferase 1
CD31/PECAM-1: cluster of differentiation 31 / platelet endothelial cell adhesion molecule 1
CEBPA: CCAAT/Enhancer Binding Protein (C/EBP) Alpha
DNMT1, 3A: DNA methyltransferases 1, 3A
ERG3: early growth response protein 3
FABP4: adipocyte protein 2, fatty acid binding protein 4
GATA2: GATA binding protein 2
HDAC2, 3: histone lysine deacetylase 2,3
IKZF1: Ikaros family zinc finger protein 1
IHH: indian hedgehog
JMJD6: jumonji domain containing 6
KAT2B, 3B: K (Lysine) acetyltransferases P300/CBP associated factor PCAF and E1A associated protein P300, respectively
JMJD2A: H3K9/36me3 lysine-specific demethylases 4A / jumonji domain containing 2A
KLF4: kruppel-like factor 4
KMT2C: lysine specific methyltransferase 2C
MBD4: methyl-CG binding domain protein 4
MYC: cMYC, proto-oncogene, transcription factor
PAI1: plasminogen activator inhibitor-1
PAXIP1: PAX interacting protein 1
PCNA: proliferating cellular nuclear antigen
PPARg: peroxisome proliferator-activated receptor gamma
PPARg2: adipocyte specific 2^nd^ isoform of peroxisome proliferator-activated receptor gamma
PRMT5: protein or histone arginine methyltransferase 5
SETDB1: SET domain bifurcated 1, histone H3K9 methyltransferase 4
SIRT1: sirtuin 1 deacetylase
SREBF1: sterol regulatory element binding transcription factor 1
TET1, 2, 3: ten-eleven translocation methylcytosine dioxygenases 1, 2, and 3
TDG: thymine-DNA glycosylase
ADSC: adipose tissue derived stem cell
BER: base excision repair pathway
DIC: differential interference phase contrast
DFAT: dedifferentiated adipocyte-derived progeny cell
FACS: fluorescence activated cell sorting
FANS: fluorescence activated nuclear sorting
FNC: fluorescence nuclear cytometry
5hmC: 5-hydroxymethylcytosine
IFM: immunofluorescence microscopy
INTACT: isolation of nuclei tagged in specific cell types
LCM: laser capture microdissection
LC-MS: Liquid Chromatography–Mass Spectrometry
MMAs: maturing and mature adipocytes
PTM: post-translational modifications
PPARg2-Pos: PPARg2-Positive
PPARg2-Neg: PPARg2-Negative
PPARg2-Med: PPARg2-Medium
qRT-PCR: quantitative real-time polymerase chain reaction
VAT, SAT, BAT: visceral, subcutaneous, and brown adipose tissue, respectively
WGBS: Whole-Genome Bisulfite Sequencing
