# Supplementary figures and images for: Subsets of Visceral Adipose Tissue Nuclei with Distinct Levels of 5-Hydroxymethylcytosine
Source: PLoS One. 2016 May 12;11(5):e0154949. doi: 10.1371/journal.pone.0154949 (PMC4865362; doi:10.1371/journal.pone.0154949)

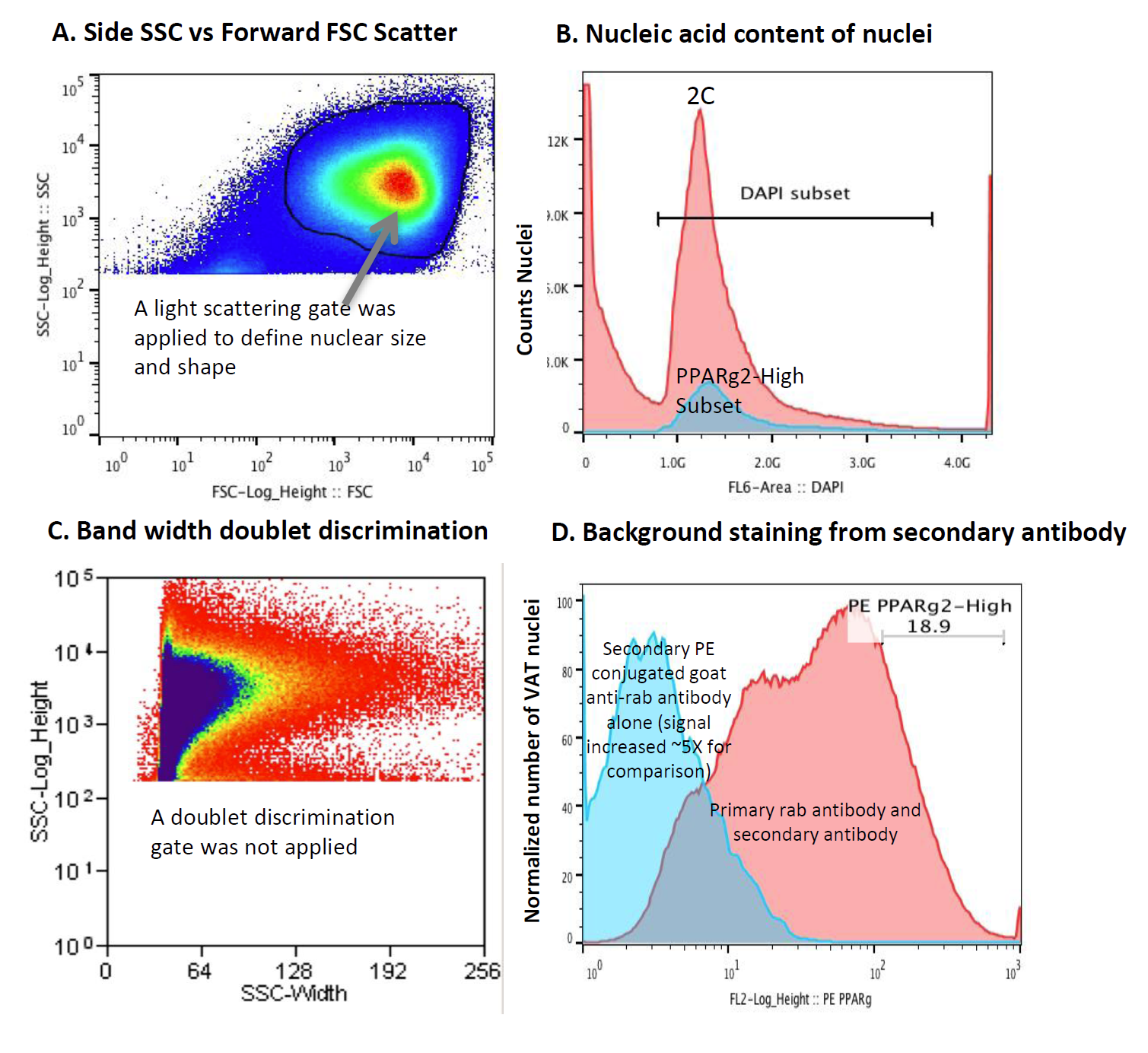

Supplement: S1 Fig — Labeled nuclei were gated for size and shape (A) and DNA content (B) to reduce the number of contaminating particles sorted. There was no significant population of doublet nuclei was detected based on side scatter band width (C). D. Background fluorescence of PE-conjugated goat anti-rabbit secondary antibody (blue) used to label rabbit anti-PPARg2 for FANS (pink) as shown in detail in Fig 3. The low background staining levels from the secondary antibody were used to define the PPARg2-Neg class of nuclei. (TIF) [file pone.0154949.s001.tif]

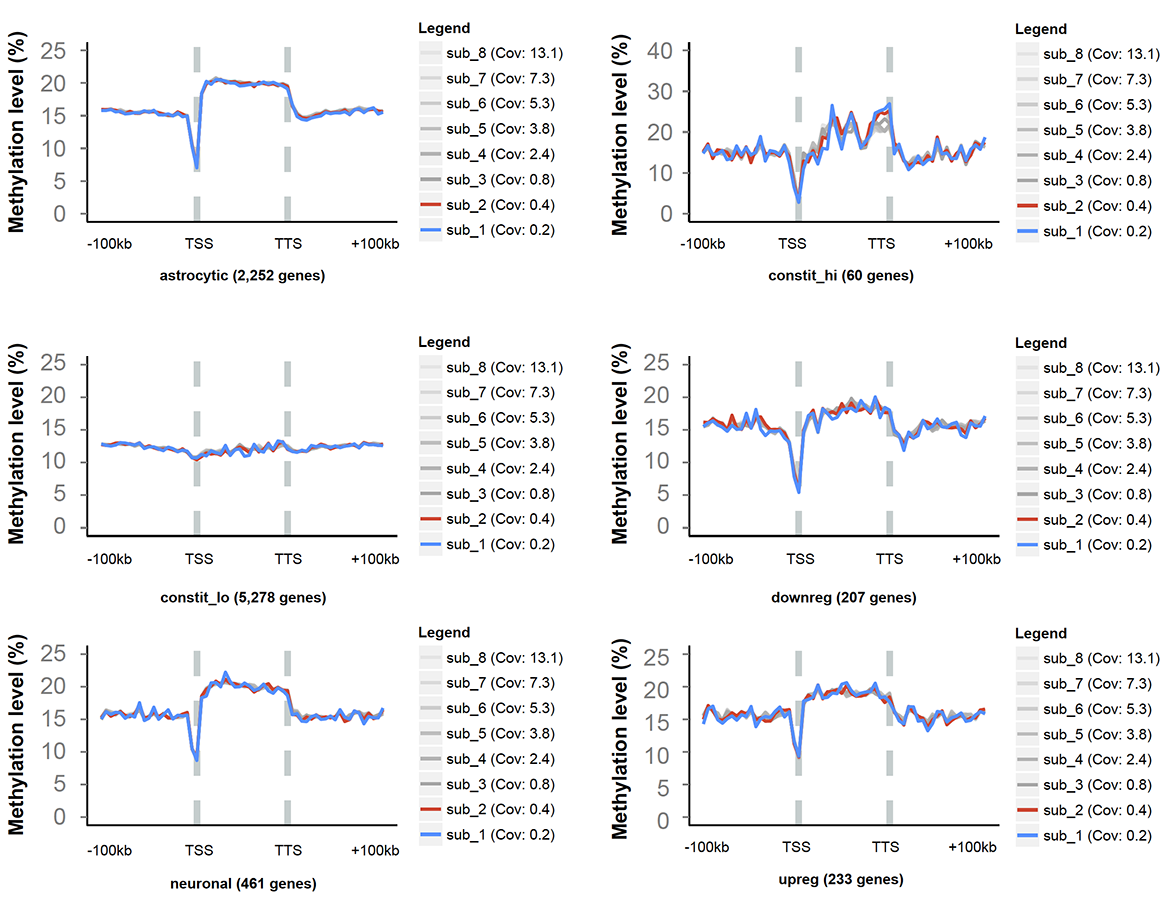

Supplement: S2 Fig — Due to high cost associated with deep coverage of the pig genome using WGBS, we chose an alternative strategy to look at 5hmC metagene plots for hundreds to thousands of genes (groups of genes). To demonstrate that this approach and our levels of coverage (i.e., 0.4X genome equivalents) is robust, we downloaded a published high-coverage 5hmC dataset including all genes in mouse frontal cortex and then subsampled number of reads ranging from 0.2X to 13X genome equivalents of coverage to plot 5hmC distribution for six gene groups. As can be seen, coverage didn’t affect the patterns of metagene plots, even for coverage as low as 0.2X. (TIF) [file pone.0154949.s002.tif]

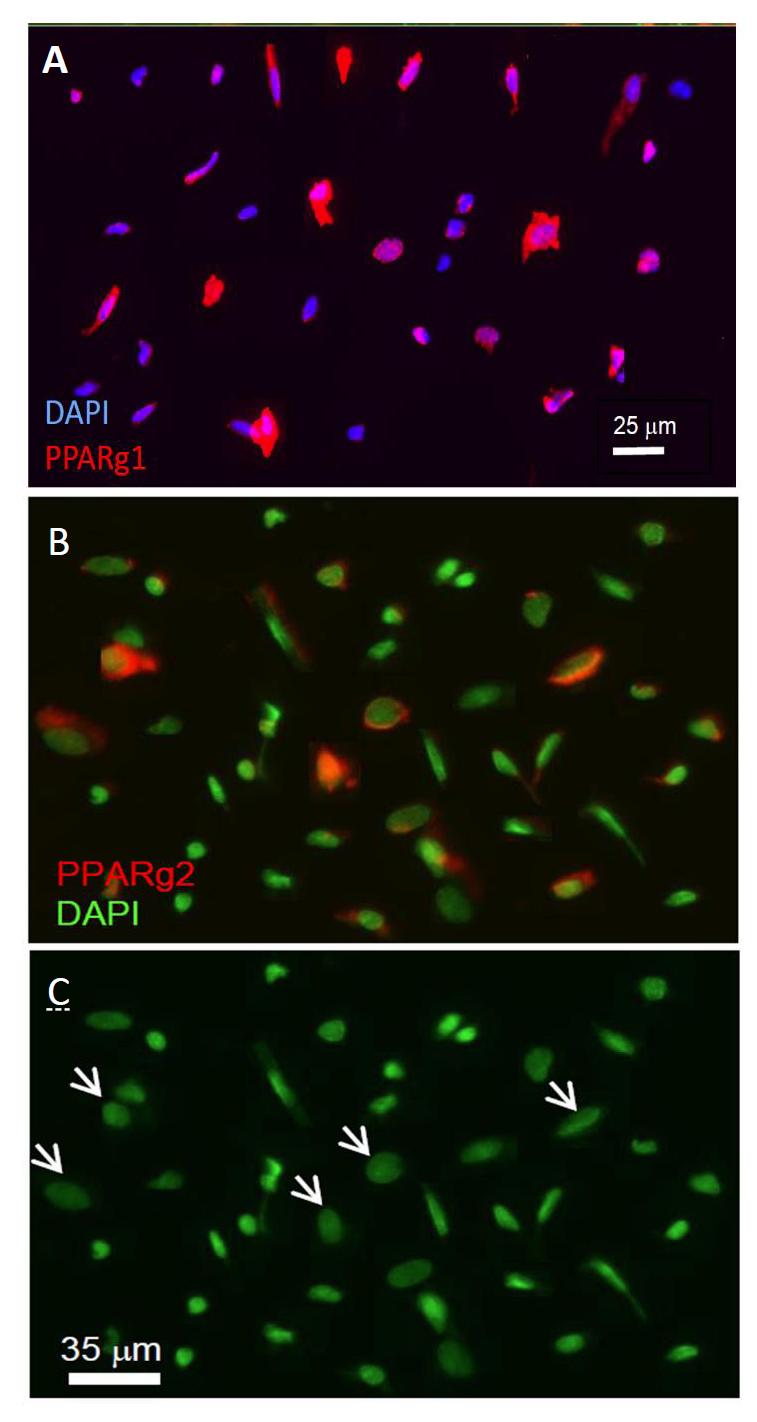

Supplement: S3 Fig — A. Nuclei were stained with DAPI for DNA (blue) and PPARg1 (red). Nuclei were stained with monoclonal antibody to PPAR-gamma (Abcam Cat.# ab70405) and then goat anti-mouse IgG conjugated with R-PE (Invitrogen Cat.# P-852) and counter stained with DAPI (blue).B. Nuclei were stained with DAPI for DNA (green) and PPARg2 (red). Nuclei were stained with polyclonal antibody to PPAR gamma 2 (Abcam Cat. # ab45036) and then Alexa fluor 633 goat anti-rabbit IgG secondary antibody (Life technologies, A21070) and counter stained with DAPI (green). (TIF) [file pone.0154949.s003.tif]

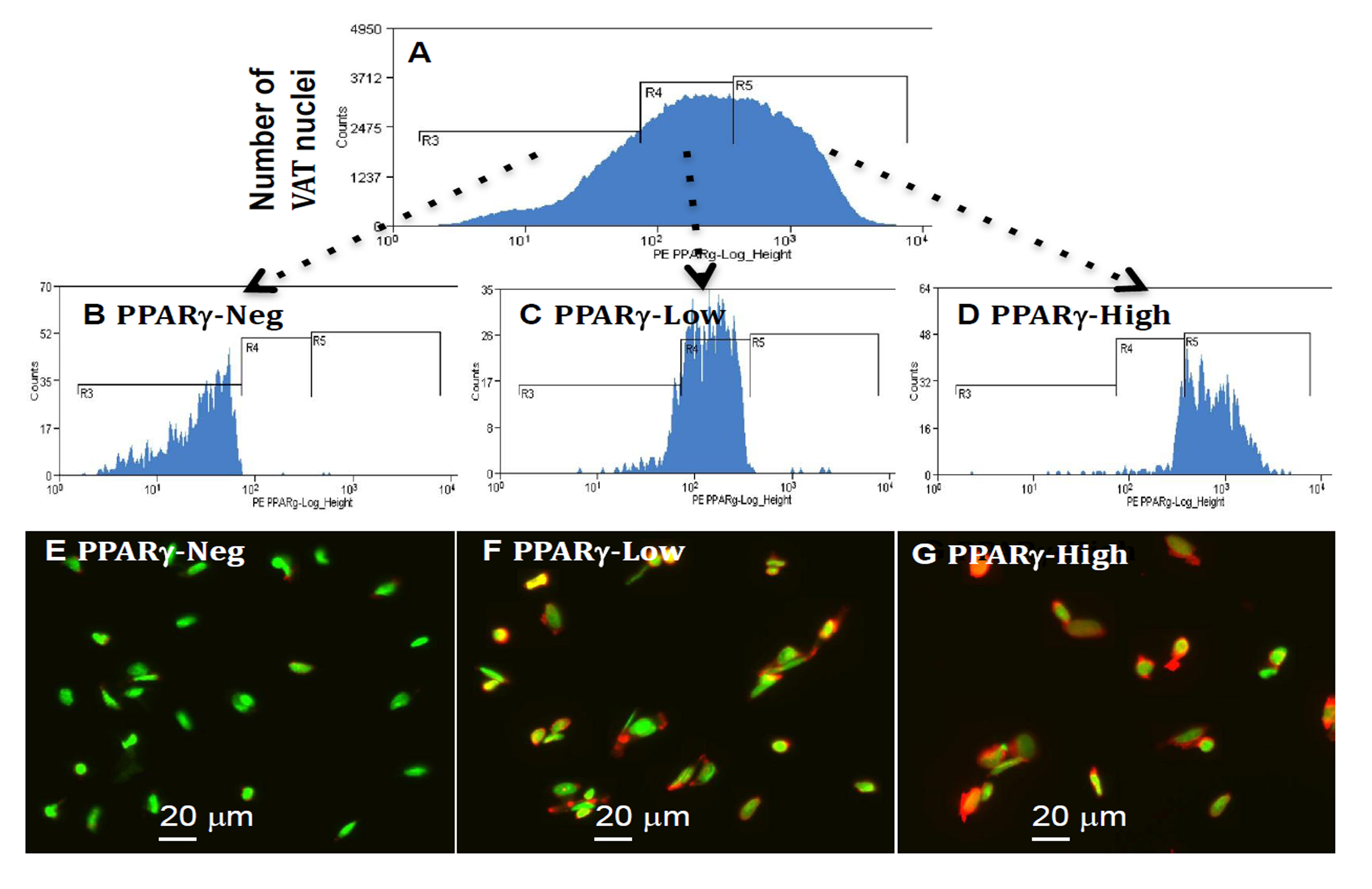

Supplement: S4 Fig — A. Histogram of PPARg1-Neg, -Low and -High stained nuclei from sorting experiment (log scale) gated for DNA content and forward and side light scattering (not shown). PPARg1 antibody (Abcam Cat.# ab70405). B, D, E. Histograms of three sorted nuclear fractions re-examined by cytometry to confirm their relative PPARg1 staining levels. E, F, G. Merged immunofluorescence microscope images of three isolated fractions of nuclei without re-staining with DAPI shown in green and PPARg1 in red. (TIF) [file pone.0154949.s004.tif]

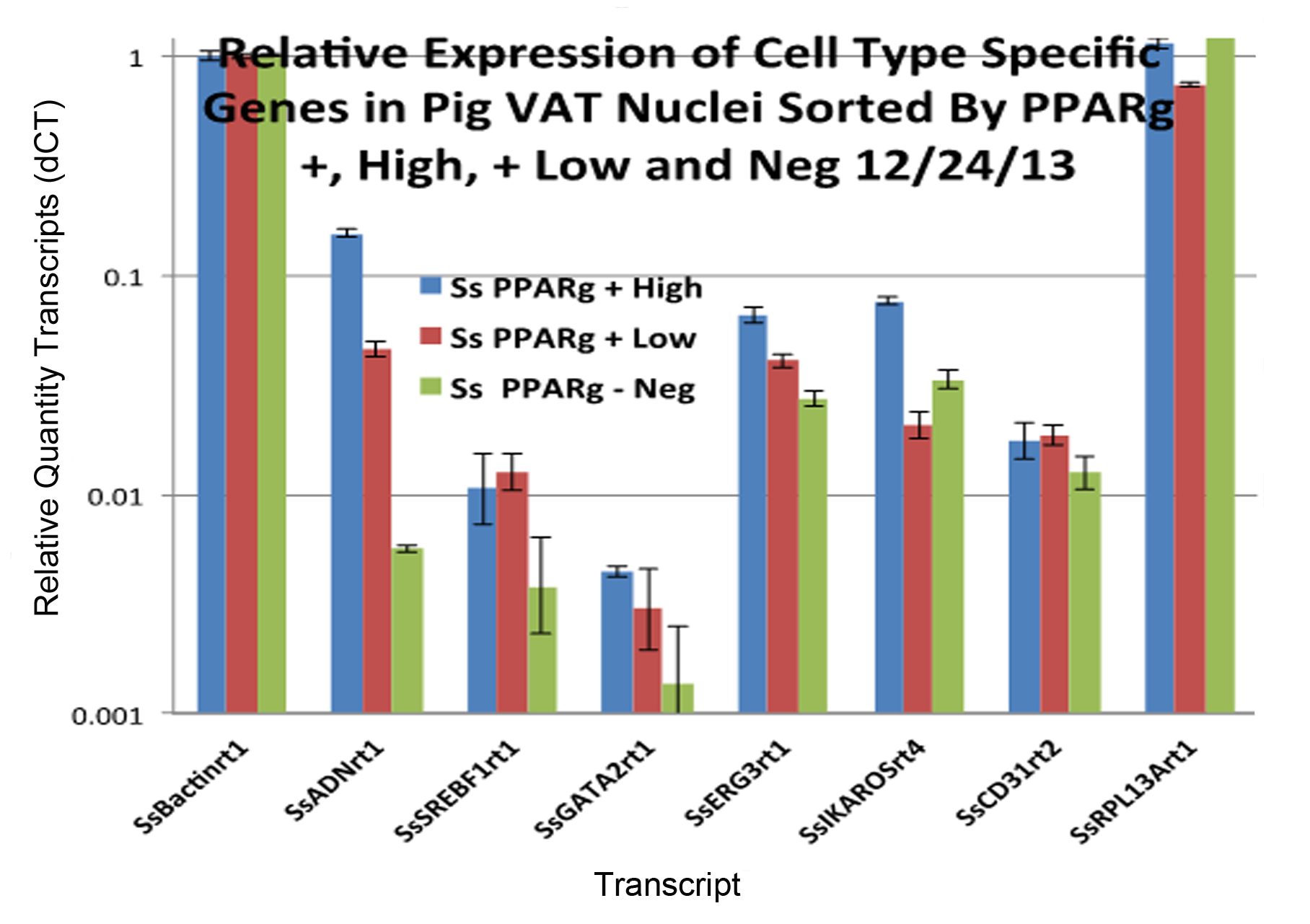

Supplement: S5 Fig — Relative quantities of marker transcripts among the four classes of VAT cell nuclei isolated by FANS were determined by qRT-PCR. Using either Beta actin or RPL13A as endogenous controls gave similar results. Cell-type-specific markers ADN, SREBF1, GATA2, ERG3, IKAROS, and CD31 were examined. The properties of maker genes and the oligonucleotide primers are described in S2 and S4 Tables, respectively. (TIF) [file pone.0154949.s005.tif]
