# Supplementary material for: Subsets of Visceral Adipose Tissue Nuclei with Distinct Levels of 5-Hydroxymethylcytosine
Source: PLoS One. 2016 May 12;11(5):e0154949. doi: 10.1371/journal.pone.0154949 (PMC4865362; doi:10.1371/journal.pone.0154949)
Supplement: S1 Table — Specific information of antibodies used in this paper were listed. (DOCX) [file pone.0154949.s006.docx]

**Table S1: Primary and Secondary Antibodies Used in this Paper**

| **Primary antibodies** | | | | | | | |
| --- | --- | --- | --- | --- | --- | --- | --- |
| **Antibody name** | **Host species** | **immunogen** | **Clonality** | | **Company** | **Catalogue or clone number** | |
| **Figure 2, 3** |  |  |  | |  |  | |
| PPAR gamma 2[1] | Rabbit | Synthetic peptide corresponding to Human PPAR gamma 2 aa 1-16. Sequence: MGETLGDSPIDPESDSC | Polyclonal | | Abcam | Cat. # ab45036 | |
| Actin [2] | Mouse | Recombinant Arabidopsis actin protein (ACT1) | Monoclonal | | Thermo Scientific | Clone. # mAbGEa | |
| Histone H3 [3] | Rabbit | Synthetic peptide within Human Histone H3 aa 100-135 (C terminal) | Polyclonal | | Abcam | Cat. # ab70550 | |
| **Figure 6** |  |  |  | |  |  | |
| 5hmC antibody[4] | Rabbit | This 5-Hydroxymethylcytosine antibody was raised against 5-hydroxymethylcytidine conjugated to KLH and recognizes 5-hydroxymethylcytosine. | Polyclonal | | Active motif | Cat.# 39769 | |
| PPARg2 ^*^ | Mouse | A DNA sequence encoding the N-terminal segment (Met 1-Thr 239) of the extracellular domain of human B7-H1(NP_054862.1) was expressed with a C-terminal polyhistidine tag. | Polyclonal | | Abeome | Clone #: 8769 | |
| **Figure S2 and S3** |  |  |  | |  |  | |
| Anti-PPAR gamma antibody [5] | Mouse | Recombinant fragment: KLIYDRCDLN CRIHKKSRNK CQYCRFQKCL AVGMSHNAIR FGRMPQAEKE KLLAEISSDI DQLNPESADL RALAKHLYDS YIKSFPLTKA KARAILTGKT T, corresponding t | Monoclonal | | Abcam | \|  \| Cat.# ab70405 \| \| --- \| --- \|   Clone #: 3A4A9,1E6A1 | |
| **Figure S5** |  |  |  | |  |  | |
| H3K4Me1[6] | Rabbit | Synthetic peptide within Human Histone H3 aa 1-100 (mono methyl K4) conjugated to Keyhole Limpet Haemocyanin (KLH). The exact sequence is proprietary. | Polyclonal | | Abcam | Cat. # ab8895 | |
| H3K9Me1[7] | Rabbit | Synthetic peptide corresponding to Human Histone H3 aa 1-100 (mono methyl K9). (Peptide available as [ab1771](http://www.abcam.cn/ab1771.html)) | Polyclonal | | Abcam | Cat. # ab8896 | |
| **Secondary Fluorescent Antibodies** | | | | | | | |
| Name | | | | Company | | | Cat. # |
| Goat pAb to Rb IgG (PE) | | | | Abcam | | | ab97070 |
| Alexa fluor 633 goat anti-rabbit IgG | | | | Life technologies | | | A21070 |
| Alexa fluor 488 goat anti-mouse IgG | | | | Life technologies | | | A11001 |

Reference:

1. Chen, Y.H., et al., *Myocyte enhancer factor-2 interacting transcriptional repressor (MITR) is a switch that promotes osteogenesis and inhibits adipogenesis of mesenchymal stem cells by inactivating peroxisome proliferator-activated receptor gamma-2.* J Biol Chem, 2011. **286**(12): p. 10671-80.

2. Kandasamy, M.K., et al., *Plant vegetative and animal cytoplasmic actins share functional competence for spatial development with protists.* Plant Cell, 2012. **24**(5): p. 2041-57.

3. Yu, P., et al., *Characterization of brain cell nuclei with decondensed chromatin.* Dev Neurobiol, 2015. **75**(7): p. 738-56.

4. Thomson, J.P., et al., *DNA immunoprecipitation semiconductor sequencing (DIP-SC-seq) as a rapid method to generate genome wide epigenetic signatures.* Sci Rep, 2015. **5**: p. 9778.

5. Wolf, D., et al., *Endothelin-1 decreases endothelial PPARgamma signaling and impairs angiogenesis after chronic intrauterine pulmonary hypertension.* Am J Physiol Lung Cell Mol Physiol, 2014. **306**(4): p. L361-71.

6. Zhang, J., et al., *Disruption of KMT2D perturbs germinal center B cell development and promotes lymphomagenesis.* Nat Med, 2015.

7. Baas, R., et al., *A novel microscopy-based high-throughput screening method to identify proteins that regulate global histone modification levels.* J Biomol Screen, 2014. **19**(2): p. 287-96.

* We validated PPARg2 mouse polyclonal antibody (Clone #: 8769) from Abeome company by staining *Sus scrofa* visceral adipose tissue (*Ss*VAT) nuclei (Figure 6), and we saw good staining signal on most of the large, decondensed *Ss*VAT nuclei.
