# Supplementary material for: Subsets of Visceral Adipose Tissue Nuclei with Distinct Levels of 5-Hydroxymethylcytosine
Source: PLoS One. 2016 May 12;11(5):e0154949. doi: 10.1371/journal.pone.0154949 (PMC4865362; doi:10.1371/journal.pone.0154949)
Supplement: S2 Table — Sense and antisense primer sequences used in this paper were listed. (DOCX) [file pone.0154949.s007.docx]

**Table S2. qRT-PCR Primers for *ss*VAT nuclei**

| **Endogenous Controls** |  |  |  |
| --- | --- | --- | --- |
| Beta actin-S1 | 5´ CTTCTAGGCGGACTGTTAGTTG  3´ | Beta actin-A1 | 5´  AGCCATGCCAATCTCATCTC 3´ |
| RPL13A -S1 | 5´ GGAGAAGGCCAAGATCCATTAC 3´ | RPL13A -A1 | 5´ CGTGAACCTGTCGATCTTCTT 3´ |
| **Figure 4** |  |  |  |
| ADN-S1 | 5´ CAGCCTCTACAAGAAGGACAAG 3´ | ADN-A1 | 5´ CTCCAGATAGAGGAGCACAGA  3´ |
| SREBF1-S1 | 5´ CGGAGGCGAAGCTGAATAAA 3´ | SREBF1-A1 | 5´ CTTCTGGTTGCTCTGCTGAA 3´ |
| GATA2-S1 | 5´ ACGACAACCACCACCTTATG 3´ | GATA2-A1 | 5´ CATGGTCAGTGGCCTGTTTA 3´ |
| ERG3-S1 | 5´ CCAGAATATCGATGGGAAGGAG 3´ | ERG3-A1 | 5´ GGTAGTGGAGATGTGACAGAAG 3´ |
| aP2-S1 | 5´ GTGTCACGGCTACCAGAATTTA 3´ | aP2-A1 | 5´ CGGGACAATACATCCAACAGAG 3´ |
| IKAROS-S4 | 5´ CGGTTGGTAAACCTCACAAATG 3´ | IKAROS-A4 | 5´ CAGCGCTCTTTATGTTCCTCTA 3´ |
| IHH-S2 | 5´ CAGCTTGCTCTCACTACAGTT 3´ | IHH-A2 | 5´ CAGGGAATCTAGCAGCATCAA 3´ |
| Klf4-S2 | 5´ TCGCCTTGCTGATTGTCTATT 3´ | Klf4-A2 | 5´ TGCCAGAGATCCTTCTTCTTTG 3´ |
| Myc-S1 | 5´ CGCTGGATTTCCTTCGGATAG 3´ | Myc-A1 | 5´ GAGTCGTAGTCGAGGTCATAGT 3´ |
| PCNA-S1 | 5´ AGGAGGAAGCAGTTACCATAGA 3´ | PCNA-A1 | 5´ CTGAGTGTGACTGTAGGAGAGA 3´ |
| **Figure 5A** |  |  |  |
| DNMT1-S2 | 5´ GCACCTCATTTGCCGAGTAT 3´ | DNMT1-A2 | 5´ CTGCAGGAACTCAACCACTATC 3´ |
| DNMT3A-S1 | 5´ TCCATAAAGCAGGGCAAAGA 3´ | DNMT3A-A1 | 5´ CATGTTGGAGACGTCGGTATAG 3´ |
| TET1-S1 | 5´ GTGTCGAGGAATCCGAAGTAAA 3´ | TET1-A1 | 5´ GAGAATAGTCCTTCACCACCAC 3´ |
| TET2-S2 | 5´ CAGGAGGGAAAGAATGCTAACT 3´ | TET2-A2 | 5´ TCCATGGAAGAGGCAGAAAC 3´ |
| TET3-S2 | 5´ CTGAAGAGCACCCATCCTTT 3´ | TET3-A2 | 5´ CTTCGGGAAACTCTGTTCTAGG 3´ |
| AICDA-S2 | 5´ GAGGCAGTTCCTCTACCAATTC 3´ | AICDA-A2 | 5´ TGAGAAGGAGGTGGCACTAT 3´ |
| **Figure 5B** |  |  |  |
| SIRT1-S2 | 5´ GTGAGGCAAAGGTTCCCTATTA 3´ | SIRT1-A2 | 5´ CCCTGAAAGTAAGACCAGTAGC 3´ |
| HDAC2-S1 | 5 CAACCTAGTGCTGTGGTGTTA 3´ | HDAC2-A1 | 5´  CACACATTTAGCGTGACCTTTG 3´ |
| HDAC3-S1 | 5´  CTGGTTACTTTCAGGGCAGTTA 3´ | HDAC3-A1 | 5´  TAGCCTTGAGAGGGAGAAGAA 3´ |
| KAT2B-S1 | 5´ CGGATGCCAAAGGAGTACAT  3´ | KAT2B-A1 | 5´  GGAAACAGATGCCACCAATAAC 3´ |
| KAT3B-S1 | 5´  GCGAAGGACTAGACTGCAAA 3´ | KAT3B-A1 | 5´ GGTTGTGGCTGTCCCATATTA 3´ |
| **Figure 5C** |  |  |  |
| KDM4A-S2 | 5´ CCTTCGACCCAGATTCGTAAA 3´ | KDM4A-A2 | 5´ ACAGAAGTCACAATCTCCAAGG 3´ |
| KMT2C-S1 | 5´ GGAGTCACCGATACACAGAATAC 3´ | KMT2C-A1 | 5´ CTGCTGGTGGAGGATGATTT 3´ |
| SETDB1-S1 | 5´ CATGTTGACCAGCTCTTTGATG 3´ | SETDB1-A1 | 5´ CTCGGTATTGTAGTCCCAGTTTAG 3´ |
| PAXIP1-S2 | 5´ CTCATGGTTTCCCATCCTCTTC 3´ | PAXIP1-A2 | 5´ CCACTCATACCACACCTTCTTG 3´ |
| ARID1A-S1 | 5´ TGAACCGCACGGATGATATG 3´ | ARID1A-A1 | 5´ GAGGCTGAAGAGGACATGTAAG 3´ |
| **Figure 5D** |  |  |  |
| JMJD6-S1 | 5´ TCAAGTGCGGTGAGGATAATG 3´ | JMJD6-A1 | 5´ CCGTAGCTGCTGTCAAAGAT 3´ |
| CARM1-S1 | 5´ GAAGGAGATTTGCACAGGATAGA 3´ | CARM1-A1 | 5´ GGACAACCACACGGTCATTA 3´ |
| PRMT5-S1 | 5´ CATCACACACAGAGGAGTACAG 3´ | PRMT5-A1 | 5´ GAAGGTCAGCACCAATTTCAAG 3´ |
| **Supplemental Figure 3** |  |  |  |
| CD31-S2 | 5´ AGGATCAGGAGGGACAGTATTA 3´ | CD31-A2 | 5´ CACTGCGATGAGTCCTTTCT 3´ |
