# Supplementary material for: Subsets of Visceral Adipose Tissue Nuclei with Distinct Levels of 5-Hydroxymethylcytosine
Source: PLoS One. 2016 May 12;11(5):e0154949. doi: 10.1371/journal.pone.0154949 (PMC4865362; doi:10.1371/journal.pone.0154949)
Supplement: S3 Table — The genome coverage achieved by TAB-seq was listed in the last column as a fraction of our coverage to Sus scrofa reference genome Sscrofa10.2 (GCA_000003025.4). (DOCX) [file pone.0154949.s008.docx]

| **Sample** | **Mapped reads** | | **Lambda** | | | | | | **pUC19 (5hmC)** | | | **Genome coverage** |
| --- | --- | --- | --- | --- | --- | --- | --- | --- | --- | --- | --- | --- |
|  |  |  | **CpG sites (5mC)** | | | **Non-CpG sites** | | |  |  |  |  |
|  | **number** | **%** | **methylated Cs** | **Total** | **%** | **methylated Cs** | **Total** | **%** | **5hmC** | **Total** | **Protection rate** |  |
| **PPARg2-High** | 7,144,386 | 36.18% | 6,818 | 699,586 | 0.97% | 14,560 | 2,076,320 | 0.70% | 3,268 | 6,244 | 52.3% | 0.41 |
| **PPARg2-Med and Low** | 6,143,977 | 35.98% | 6,962 | 704,566 | 0.99% | 15,146 | 2,096,005 | 0.72% | 2,916 | 5,767 | 50.6% | 0.35 |
| **PPARg2-Neg** | 6,980,655 | 36.45% | 6,854 | 728,707 | 0.94% | 14,490 | 2,159,023 | 0.67% | 3,245 | 6,698 | 48.4% | 0.40 |

Table S3: TAB-seq Analysis Summary

Provided in the table are the metrics related to the TAB-seq analysis. The genome coverage achieved by our TAB-seq meta-analysis is listed in the last column as a fraction of our coverage to the *Sus scrofa* reference genome *Sscrofa*10.2.
