# Supplementary material for: Subsets of Visceral Adipose Tissue Nuclei with Distinct Levels of 5-Hydroxymethylcytosine
Source: PLoS One. 2016 May 12;11(5):e0154949. doi: 10.1371/journal.pone.0154949 (PMC4865362; doi:10.1371/journal.pone.0154949)
Supplement: S4 Table — The symbol, full name and description of the genes assayed in this paper were listed. (DOCX) [file pone.0154949.s009.docx]

**Table S4: Summary information and references on the properties of the marker genes being assayed**

| Symbol | Full Name | Description |
| --- | --- | --- |
| ACTB/Bactin | Beta actin | This gene encodes one of six different actin proteins. Actins are highly conserved proteins. This actin is a major constitute of the contractile apparatus and one of the two nonmuscle cytoskeletal actins. |
| ADIPOQ | Adiponectin | This gene is expressed in adipose tissue exclusively. It is an important adipokine involved in the control of fat metabolism and insulin sensitivity. It is expressed in white, beige, and brown adipose tissue. |
| AICDA | Activation-induced cytidine deaminase | This gene encodes a RNA-editing deaminase that is a member of the cytidine deaminase family. |
| FABP4 | Adipocyte protein 2, fatty acid binding protein 4 | This gene encodes the fatty acid binding protein found in adipocytes. Its roles include fatty acid uptake, transport, and metabolism. After 10 days of 3T3-L1 differentiation, aP2 was induced 150-fold in differentiated adipocytes. |
| ARID1A | AT rich interactive domain 1A | This gene encodes a member of the SWI/SNF family, which have helicase and ATPase activities and are able to regulate transcription of certain genes by altering the chromatin structure around those genes. It is required for adipogenesis from ES cells. Controls Sox2, Utf1, and Oct4 expressin. |
| CARM1 | Coactivator-associated arginine methyltransferase 1 | This gene belongs to the protein arginine methyltransferase (PRMT) family. It methylates histone H3 at Arg-17(H3R17me), forming H3R17me2, leading to activate transcription via chromatin remodeling. |
| CD31/PECAM-1 | Cluster of differentiation 31 | This protein is found on the surface of platelets, monocytes, neutrophils, and some types of T-cells, and makes up a large portion of endothelial cell intercellular junctions. |
| DNMT1 | DNA methyltransferase 1 | DNMT1 has a role in the establishment and regulation of tissue-specific patterns of methylated cytosine residues. DNA methyltransferases preserve the  methylation pattern of the parent cell during mitosis by methylating the nonconserved strand during replication (Okano et al., 1999). |
| DNMT3A | DNA methyltransferase 3A | DNMT3A is required for genome-wide de novo methylation and is essential for the establishment of DNA methylation patterns during development. |
| ERG3 | Early growth response protein 3 | This gene encodes a transcriptional regulator that belongs to the EGR family of C2H2-type zinc-finger proteins. It plays a role in muscle development, lymphocyte development, endothelial cell growth and migration. It is a sterol C5-desaturase involved in cholesterol biosynthesis (Acimovic et al., 2011). |
| GATA2 | GATA binding protein 2 | It promotes the differentiation of MSCs into adipocytes (Kamata et al., 2014). |
| HDAC2 | Histone lysine deacetylase 2 | It is responsible for the deacetylation of lysine residue at the N-terminal regions of core histones. It plays an important role in transcription regulation, cell cycle progression and development. |
| HDAC3 | Histone lysine deacetylase 3 | It plays a critical role in transcription regulation, cell cycle progression, and development. It has deacetylase activity and represses transcription when tethered to a promoter. |
| IKZF1 | Ikaros family zinc finger protein 1 | The gene encodes a transcription factor that belongs to the family of zinc-finger DNA-binding proteins associated with chromatin remodeling. The expression of this protein is restricted to the hemo-lymphopoietic system, and it functions as a regulator of lymphocyte differentiation. |
| IHH | Indian Hedgehog | IHH is present in preadipocytes and its expression decreases upon differentiation. As mesenchymal cells commit into adipocytes they lose their ability to express Ihh (Cousin, Dani et al. 2006). |
| JMJD6 | Jumonji Domain Containing 6 | This gene encodes a nuclear protein with a JmjC domain. It is a histone arginine demethylase. |
| KAT2B | Lysine acetyltransferase 2B or P300/CBP-associated factor | It is a histone acetyltransferase to promote transcription activation. It has significant histone acetyltransferase acticity with core histones (H3 and H4), and also with nucleosome core particles. |
| KAT3B | Lysine acetyltransferase P300 | It functions as histone acetyltransferase that regulate transcription via chromatin remodeling. |
| KDM4A | Lysine-specific demethylase 4A | Histone demethylase that specifically demethylates lysine 9 and lysine 36 residues of histone H3. KDM4A generates H3K9Me from the di-and tri-methylated forms. |
| KLF4 | Kruppel-like factor 4 | It regulates the expression of key transcription factors during embryonic development. Plays an important role in maintaining embryonic stem cells, and in preventing their differentiation. |
| KMT2C | Lysine specific methyltransferase 2C | It is a histone lysine methyltransferase that methylate lysine 4 of histone H3. H4K4 methylation represents a specific tag for epigenetic transcription activation. |
| MBD4 | Methyl-CpG biding domain protein 4 | Mismatch-specific DNA N-glycosylase involved in DNA repair. It has thymine glycosylase acticity and is specific for G:T mismatches within methylated and unmethylated CpG sites. It can also remove uracil or 5-fluorouracil in G:U mismatches. |
| MYC | Proto-oncogene C-Myc | The protein encoded by this gene is a multifunctional, nuclear phosphoprotein that plays a role in cell cycle progression, apoptosis and cellular transformation. |
| PAXIP1 | PAX Interacting (With Transcription-Activation Domain) Protein 1 | Histone Methylation Regulator PTIP Is Required for *PPAR*γ and *C/EBP*α Expression and Adipogenesis. PTIP is a protein that associates with histone H3K4 methyltransferases. |
| PCNA | Proliferating cellular nuclear antigen | PCNA acts as a homotrimer and helps increase the processivity of leading strand synthesis during DNA replication. |
| PPARg | Peroxisome proliferator-activated receptor gamma | This gene encodes a member of the peroxisome proliferator-activated receptor (PPAR) subfamily of nuclear receptors. PPAR gamma is a regulator of adipocyte differentiation. |
| PPARg2 | Adipocyte specific 2^nd^ isoform of peroxisome proliferator-activated receptor gamma | N-terminal domain isoform of PPARg. It has highest expression in adipose tissue than any other tissues, like muscle, spleen, heart and liver. It is more adipocyte specific. It is a nuclear receptor that binds peroxisome proliferators such as fatty acids. Once activated by a ligand, the nuclear receptor binds to DNA specific PPAR response elements (PPRE) and modulates the transcription of its target genes. |
| PRMT5 | Protein arginine methyltransferase 5 | This gene encodes a histone arginine methyltransferase 5.  PRMT5 plays a high-level causal role in adipogenesis, as small RNA silencing PRMT5 expression blocks PPARg2 expression and efficiently blocks the differentiation of 3T3-L1 preadipocytes into mature lipid-containing adipocytes (LeBlanc et al., 2012).  PRMT5 promotes gene expression of PPARgamma2 and its target genes during adipogenesis. |
| SETDB1 | SET domain bifurcated 1 | This gene encodes a histone lysine methyltransferase.  SETDB1 renders chromatin inactive through H3K9 methylation to shut off PPARg. SETDB1 is intriguing as this is the first methyl transferase for which its enzymatic function has been linked to a cell fate decision through epigenetic re gulation in response to extracellular stimulation (Takada 2009). |
| SIRT1 | Sirtuin 1 deacetylase | It is also known as NAD-dependent deacetylase sirtuin-1. SIRT1 is a histone deacetylase. |
| SREBF1 | Sterol regulatory element binding transcription factor 1 | Transcriptional activator required for lipid homeostasis. It is expressed in adipocytes, fatty liver cells. It regulates glucose metabolism and fatty acid and lipid production and its expression is regulated by insulin. |
| TET1, 2, 3 | Ten-eleven translocation methylcytosine dioxygenases 1, 2, and 3 | TETs catalyze the conversion of 5-methylcytosine (5mC) to 5-hydroxymethylcytosine (5hmC) and subsequent conversion 5hmC into 5-formylcytosine (5fC) and 5-carboxylcytosine (5caC), and plays a key role in active DNA demethylation. |
| TDG | Thymine-DNA Glycosylase | TDG plays a key role in active DNA demethylation. It recognizes 5fC and 5caC and mediates their excision through base-excision repair to install an unmethylated cytosine. |

Reference inserted by endnote (Ping’s endnote library):

Cousin, W., C. Dani and P. Peraldi (2006). "Inhibition of the anti-adipogenic Hedgehog signaling pathway by cyclopamine does not trigger adipocyte differentiation." Biochem Biophys Res Commun **349**(2): 799-803.

References waiting to be inserted:

**Acimovic, J., Korosec, T., Seliskar, M., Bjorkhem, I., Monostory, K., Szabo, P., Pascussi, J.M., Belic, A., Urleb, U., Kocjan, D., and Rozman, D.** (2011). Inhibition of human sterol Delta7-reductase and other postlanosterol enzymes by LK-980, a novel inhibitor of cholesterol synthesis. Drug Metab Dispos **39**, 39-46. <http://www.ncbi.nlm.nih.gov/pubmed/20952551>.

**Kamata, M., Okitsu, Y., Fujiwara, T., Kanehira, M., Nakajima, S., Takahashi, T., Inoue, A., Fukuhara, N., Onishi, Y., Ishizawa, K., Shimizu, R., Yamamoto, M., and Harigae, H.** (2014). GATA2 regulates differentiation of bone marrow-derived mesenchymal stem cells. Haematologica **99**, 1686-96. <http://www.ncbi.nlm.nih.gov/pubmed/25150255>.

**LeBlanc, S.E., Konda, S., Wu, Q., Hu, Y.J., Oslowski, C.M., Sif, S., and Imbalzano, A.N.** (2012). Protein arginine methyltransferase 5 (Prmt5) promotes gene expression of peroxisome proliferator-activated receptor gamma2 (PPARgamma2) and its target genes during adipogenesis. Mol Endocrinol **26**, 583-97. <http://www.ncbi.nlm.nih.gov/pubmed/22361822>.
